# Peer review of health research funding proposals: A systematic map and systematic review of innovations for effectiveness and efficiency

**DOI:** 10.1371/journal.pone.0196914

**Published:** 2018-05-11

**Authors:** Jonathan Shepherd, Geoff K. Frampton, Karen Pickett, Jeremy C. Wyatt

**Affiliations:** Wessex Institute, Faculty of Medicine, University of Southampton, Southampton, United Kingdom; Lancaster University, UNITED KINGDOM

## Abstract

**Objective:**

To investigate methods and processes for timely, efficient and good quality peer review of research funding proposals in health.

**Methods:**

A two-stage evidence synthesis: (1) a systematic map to describe the key characteristics of the evidence base, followed by (2) a systematic review of the studies stakeholders prioritised as relevant from the map on the effectiveness and efficiency of peer review ‘innovations’. Standard processes included literature searching, duplicate inclusion criteria screening, study keyword coding, data extraction, critical appraisal and study synthesis.

**Results:**

A total of 83 studies from 15 countries were included in the systematic map. The evidence base is diverse, investigating many aspects of the systems for, and processes of, peer review. The systematic review included eight studies from Australia, Canada, and the USA, evaluating a broad range of peer review innovations. These studies showed that simplifying the process by shortening proposal forms, using smaller reviewer panels, or expediting processes can speed up the review process and reduce costs, but this might come at the expense of peer review quality, a key aspect that has not been assessed. Virtual peer review using videoconferencing or teleconferencing appears promising for reducing costs by avoiding the need for reviewers to travel, but again any consequences for quality have not been adequately assessed.

**Conclusions:**

There is increasing international research activity into the peer review of health research funding. The studies reviewed had methodological limitations and variable generalisability to research funders. Given these limitations it is not currently possible to recommend immediate implementation of these innovations. However, many appear promising based on existing evidence, and could be adapted as necessary by funders and evaluated. Where feasible, experimental evaluation, including randomised controlled trials, should be conducted, evaluating impact on effectiveness, efficiency and quality.

## Introduction

Peer review is a key element of quality assurance in academic research. [1] It is used to reassure research funders that research proposals are of the highest scientific merit and that funded research is appropriate to policy and practice needs. Peer review is also employed at later stages of the research lifecycle to improve the scientific credibility of research outputs, such as articles in academic journals. There is a need to ensure that peer review is effective and efficient, to support the production of high quality research across the sciences. [2]

However, there are challenges. Many research funders are facing increasing budgetary pressure and need to ensure that peer review, alongside other aspects of research management, is efficient in time and costs. [3] Peer review has also been subject to criticisms calling into question its validity and usefulness as a process for identifying the ‘best’ scientific research. [4, 5] For example, peer review can be time consuming and therefore expensive, and funders often make substantial efforts to identify and recruit appropriate reviewers and obtain sufficient feedback from them in a timely manner. [3] Researchers typically spend several weeks or months preparing a proposal [6] and each year hundreds of years’ worth of total reviewers’ time are used by individual research councils, [7, 8] which equates to tens of millions of pounds in salary costs. [6] The value of this investment is diminished if peer review is unable to identify good quality proposals that ultimately will have a high impact on policy, practice and science.

Despite the effort involved, it has been argued that peer review leads to inconsistent funding decisions which may be no better than chance decisions in selecting the best proposals. [9] In some cases, however, good correlations have been reported between peer review scores and the estimated scientific impact of the funded proposals. [10] In addition to concerns about the effort involved, peer review has been criticised as being biased, which may reflect a disproportionate influence of individual reviewers’ preferences [11] or conflicts of interest. [2] Common concerns are that peer review can be associated with gender bias, or institutional bias, may penalise inexperienced research applicants, and that traditional peer review systems used by major funding agencies tend to be conservative, rejecting innovative or ‘high-risk’ research proposals. [12] Criticism has also been made of the ‘black box’ nature of peer review, and attempts have been made to better understand the social and cultural processes by which multi-disciplinary academic funding panels discuss applications, define academic excellence and make funding decisions. [13]

Nonetheless, peer review remains a significant aspect of research commissioning, and some funding agencies have attempted to address the criticisms. For example, the US National Institutes of Health and UK Research Councils (among others) have studied their peer review practices to identify opportunities for improvement. Funders are increasingly exploring improvements to peer review processes and methods, or alternatives to peer review itself. [4, 14] These include using open rather than blinded review, use of digital technology to discuss proposals rather than face-to-face meetings, testing new proposal scoring methods, and introducing shorter proposal forms and expedited review processes.

Given the costs of peer review and its centrality in ensuring the quality of research, there is a need to map alternative approaches to peer review and assess their impact in addressing some of the criticisms made. There have been few previous systematic reviews in this area. A Cochrane systematic review [15] assessed the impact of a variety of peer review processes on the quality of funded research, identified from the health literature. The review included 10 studies, conducted in a range of countries. Overall, the authors concluded that the quality of the evidence base was limited and that there is a strong need for experimental studies to examine the impact of different peer review processes on the quality of funded research. Given that the literature searches were carried out in 2002 this review is now very out-of-date. This underlines the need for an up-to-date comprehensive review of the evidence.

The question this project set out to investigate was: What is the research evidence on methods and processes for timely, efficient and good quality peer review of research funding proposals in health? The purpose was to make recommendations which could then be made to research funders about useful methods that could potentially be adopted, as well as identifying where further research into peer review of health research proposals is needed. This project was one of a number of complementary research projects conducted within a UK health research funder, the National Institute for Health Research (NIHR), to investigate potential improvements to the process of the peer review of funding applications.

## Methods

A two-stage evidence synthesis was conducted comprising: (1) systematic mapping of the key characteristics of the evidence base, followed by: (2) a systematic review of a sub-set of studies on a particular area of relevance prioritised from the map by stakeholders. This is a flexible and pragmatic approach to evidence synthesis that has been successfully applied in a number of published systematic reviews of complex health and education interventions as a means of characterising the evidence base to facilitate a policy-relevant, stakeholder-informed synthesis. [16–20] Stakeholder involvement in systematic reviewing, including the setting of the scope and the research questions, has become increasingly important in evidence-informed health in recent years. [21] The intended methods were described in a research protocol which was circulated amongst NIHR stakeholders for comment before being finalised (S1 Protocol). This was not pre-published in the PROSPERO systematic review repository as it did not include a health outcome, so was ineligible.

### Systematic map

#### Literature searching

A comprehensive search for relevant literature was undertaken by an experienced health information specialist. A draft search strategy was created, piloted, and revised before implementation (S1 Appendix). The following electronic bibliographic databases were searched using the same strategy adapted for each database as necessary (the host platforms used are indicated in brackets): Medline (Ovid); MEDLINE In-Process & Other Non-Indexed Citations (Ovid); Embase (Ovid); The Cochrane Library (comprising the Cochrane Database of Systematic Reviews; Cochrane Central Register of Controlled Trials (CENTRAL); and Database of Abstracts of Reviews of Effects); Psychinfo (Ebsco); Social Sciences Citation Index (Web of Science); and Delphis (a University of Southampton Library database). Database searches were conducted during May-June 2016. We also searched the internet sites of international health research funders and health charities (S1 Protocol) during June-July 2016. Reference lists of a random sample of 25% of articles included in the systematic map, and of all studies included in the systematic review were searched to check that relevant studies had not been missed. All references identified from electronic databases were imported into an Endnote reference management library for storage, removal of duplicates, retrieval of the full text versions, and eligibility screening.

#### Systematic map eligibility criteria

To be included in the map the references needed to report a research study, of any design, investigating any aspect of the peer review of health research funding application process. Systematic reviews were also permitted but commentary, opinion and editorial articles were excluded. For this project health research was defined broadly to include research into health and social care, public health, and health promotion. References reporting investigations into the peer review of research outputs were not eligible unless they also reported an investigation into the peer review of funding applications. Study inclusion was limited to articles published in the English language. Before being fully implemented, the inclusion criteria were piloted by two reviewers independently on a sample of titles and abstracts which were published in 2015–2016 and retrieved by the literature search.

Each title and abstract was screened independently by two reviewers (JS, GF or KP) with extensive experience of systematic reviewing. If agreement between reviewers could not be reached a third reviewer was consulted. The full text versions of references deemed potentially relevant on checking their titles and abstracts were retrieved for further screening. All full text articles were screened by one reviewer and checked by a second. A third reviewer was consulted in cases of disagreement.

#### Systematic map coding

A draft set of keywords was devised and agreed by the research team (JS, GF, KP, JW) to describe the key characteristics of the studies relevant to this project. Terms were created for aspects such as: the scope of the studies; the study population (e.g. researchers, health professionals); the study design (e.g. experimental, observational); the study context (e.g. country; type of research funder); and study measures, including outcome and process measures. The keywords did not, however, characterise the results of studies as this was the purpose of the subsequent systematic review.

The draft keyword list was pilot-tested on a subset of 13 studies from the map, [6, 10, 22–32] to ensure validity and consistency of application between reviewers. The draft list was also circulated for general comment amongst relevant stakeholders from a working group on peer review as part of the NIHR’s strategic priority project ‘Push the Pace 2’ (which aims to establish a proportionate peer review system for research proposals). The final version of the keyword list is provided in a Microsoft Excel worksheet (S1 Database). All included full-text articles reporting an individual study were grouped and read together and the keywords which were applicable to the study were coded in the worksheet by one reviewer. A random sample of 20% of the studies (n = 16/83) was checked by a second reviewer to ensure reliability and comprehensiveness. The level of reliability between reviewers was considered sufficient, since fewer than 2% of the checked data cell entries in the map worksheet required amendments, which were relatively minor.

Upon completion of the keywording the applied coding was analysed within the database to generate frequencies and cross-tabulations of keywords, permitting an overview of the characteristics of the evidence. The research team met to discuss the results and to identify potential sub-sets of studies grouped by sets of keywords reflecting a particular issue or theme (‘scenario’) for potential inclusion in the systematic review.

#### Stakeholder topic prioritisation

Based on the peer review issues reported in the systematic map e.g. bias, quality assurance, efficiency, and study context (e.g. country, type of research funder), and the study outcomes and process measures (e.g. funding decisions made, impact of the funded research), the research team identified three contrasting evidence scenarios for potential systematic review. The scenarios were devised to be relevant to stakeholders involved in research commissioning and management.

The three scenarios were tabulated and emailed to the NIHR Working Group on peer review prior to a face-to-face meeting to discuss the scenarios. The meeting was attended by three of the current authors and 11 members of the working group, who represented all of the different NIHR research commissioning centres. Each scenario was described and discussed in turn and stakeholders were given the opportunity to ask the research team for more information about the scenario and pertinent evidence from the map.

Following the meeting a summary of the discussion was circulated to the NIHR working group members not present at the meeting to seek any additional comments. There was no disagreement from any of these other group members on the prioritised scenario. Further detail on the stakeholder topic prioritisation process is reported in S2 Appendix.

### Systematic review

Following the stakeholder consultation exercise the prioritised scenario question for the systematic review was: “Which innovations can improve the efficiency and/or effectiveness of the peer review of health research proposals?”

A set of inclusion criteria for the systematic review was drafted to reflect this research question. The final criteria were: 1) Primary outcome evaluation studies or systematic reviews on the peer-review of research funding proposals in health published after 2005 (N.B. Systematic reviews were to be included as a source of references only); 2) Any peer review system structure innovation, with the exception of ranking or scoring of grant proposals (these were not considered relevant by the stakeholders); 3) At least one outcome measure relating to the efficiency of peer review (e.g. time required by peer reviewers; administrative costs of peer review; level of agreement between reviewers) and/or the effectiveness of peer review (e.g. ability of peer review to inform funding decisions; quality of the peer review process; scientific quality of the funded research and its impact on policy, practice and science).

The inclusion criteria were applied to the full text articles of studies already located in the systematic map. One reviewer applied the criteria and a second checked their decision, with any disagreements resolved through discussion. Studies meeting the criteria underwent data extraction and critical appraisal using a template devised for this study.

Due to the diverse range of potentially eligible studies, a number of critical appraisal instruments were considered for use. Any randomised controlled trials (RCTs) identified were to be appraised using the Cochrane risk of bias tool. [33] A modification to these criteria for non-randomised studies by the Cochrane Effective Practice and Organisation of Care (EPOC) group was also planned. However, this was not subsequently applied to any of the included studies due to the nature of their designs (see ‘Results‘ below). Few existing instruments were considered appropriate for critically appraising the included studies and therefore we undertook a narrative appraisal of the quality of each study, commenting on key aspects of data collection and analysis and threats to internal validity. Data extraction and critical appraisal was performed by one reviewer (JS or GF) and checked by a second with any disagreements resolved through discussion.

Given the heterogeneous nature of the included studies (the studies differed considerably in their designs and characteristics) it was not considered appropriate to conduct meta-analysis. A narrative synthesis was therefore conducted.

## Results

### Systematic map results

**Fig 1:**
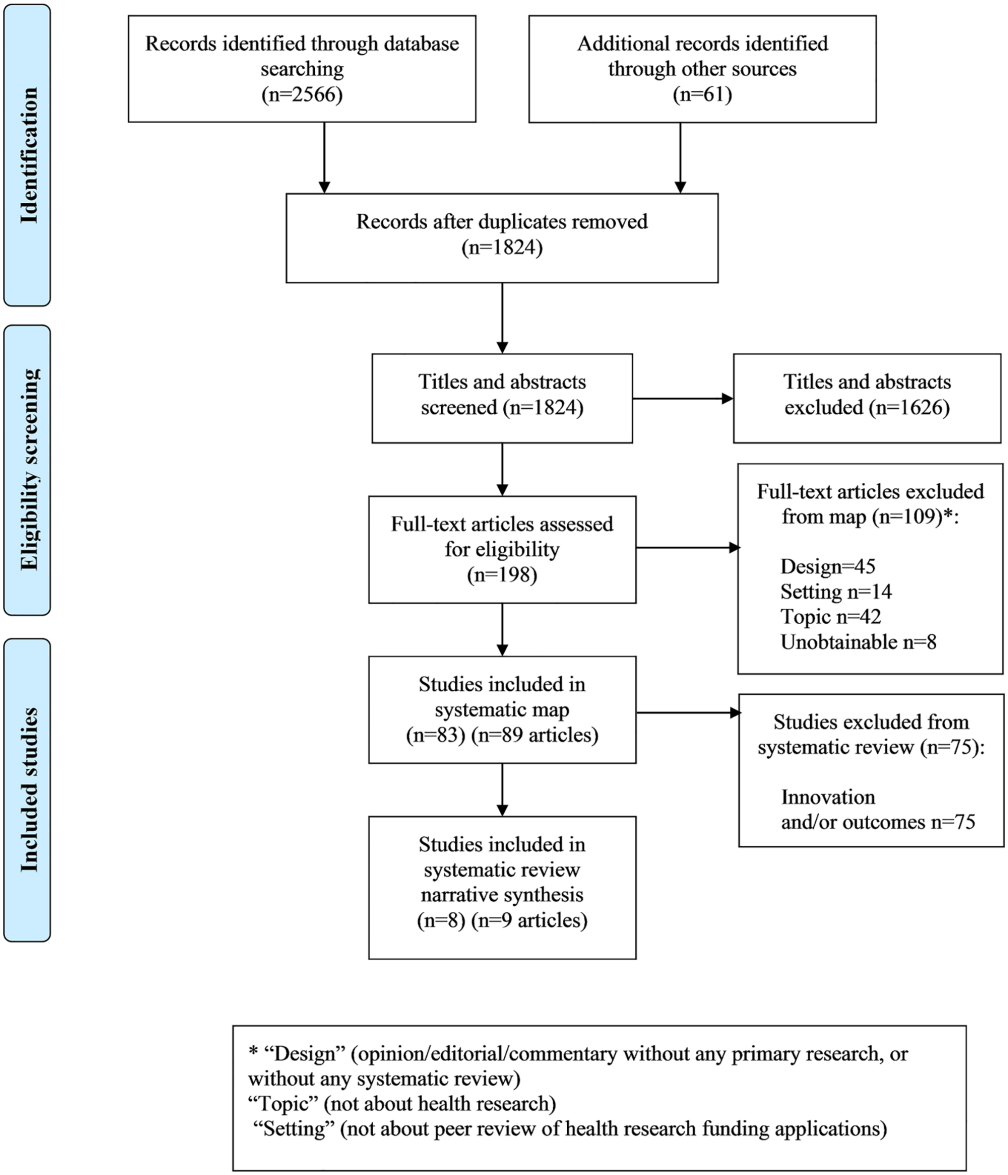
Preferred Reporting Items for Systematic Reviews and Meta-Analyses (PRISMA) flowchart.

A total of 1824 titles and abstracts was screened, and 198 of these were further screened as full text articles (Fig 1). The rate of agreement between the two reviewers at full-text screening was 90%, with 10% of the decisions requiring further discussion or referral to a third reviewer to reach a final decision. A total of 83 studies (described in 89 publications) met the inclusion criteria for the systematic map. [3, 6, 9, 10, 12, 15, 23–30, 32, 34–104] (S1 Database).

Most studies (72%) were published from 2005 onwards (49% from 2010 onwards). Fifteen countries were represented, with 49% of studies having been conducted in the USA. Other locations included Europe (23%, most frequently in Germany and the UK [each 6%]); Canada (11%), and Australia (9%). Of the study types, 61% were observational; 31% were based on surveys, interviews or focus groups; and 7% were experimental (of which 3 studies [4%] were randomised). In the majority of studies (73%) the setting was a national research council (e.g. the US National Institutes of Health; NIH). A smaller proportion of studies were based in charities or local funders. In around one third of the studies the peer reviewers were academics and/or health professionals, and in 10% they were lay people. In the majority of studies, however, the professional status of the peer reviewers was not reported. In some studies the peer reviewers were external to the funder and its funding decision panel, whilst in other cases the reviewers were also involved in making funding decisions. In many studies the extent of the reviewer’s role (e.g. funding panel member) was not clearly defined.

A variety of peer review issues have been studied. We categorised these as relating to the process and structure of a peer review system, such as: scoring/ranking methods (12%); configurations of reviewers (e.g. the number needed or expertise required) (12%); or methods for identifying peer reviewers (7%); and peer reviewer processes, such as: bias in peer review (20%); predictive ability of peer review to identify research projects that will ultimately be successful (22%); consistency in reviewing scoring/judgements between reviewers (18%); and stakeholder opinions on the peer review process (30%).

### Systematic review results

Eight studies met all the inclusion criteria for the systematic review and are summarized in Table 1. These evaluated a broad range of innovations which can be categorised as: shortening of grant proposals (alongside other peer review simplifications); [6, 23, 29] videoconferencing or teleconferencing approaches; [47, 60, 100] a Delphi consensus approach; [27], a video training module for peer reviewers; [95] and involvement of patients and other care-giving stakeholders to improve peer review. [57] Table 2 provides our critical appraisal of each study and Table 3 describes features of the studies which relate to their generalizability. S1 Table provides tabulated details of the study results, ordered by outcome and process measure. A structured narrative description of the methods and results of each study follows.

**Table 1:** Overview of peer review (PR) studies included in the systematic review.

Study ID, country	Innovation intervention(s)	Comparator(s)	Efficiency outcome(s)	Effectiveness outcome(s)	Process measures	Study design
Barnett et al. 2015 [23], Australia	Short proposal with simplified scoring and accelerated PR	None	Proposal preparation time; PR time	Funding outcome	Applicants’ views (summary only)	Observational; part of a quality improvement evaluation
Fleurence et al. 2014 [57], USA	Process to engage patients and other stakeholders in PR	None	Reviewer agreement	Funding outcome	Reviewers’ views	(Unclear whether retrospective) analysis of PCORI inaugural funding round
Gallo et al. 2013 [60], Carpenter et al. 2015 [47], USA	Teleconference PR panels; Videoconferencing panels (pilot test)	Face-to-face PR panels	PR time; Reviewer agreement	Funding outcome (assumption-based)	Reviewers’ views	Retrospective analysis
Herbert et al. 2015 [6], Australia	2 simplified face-to-face assessments: (1) 7-reviewer panel assessed 9-page proposal + applicant track record; (2) 2-reviewer panel assessed 9-page proposal only	Standard face-to-face assessment: 12-reviewer panel assessed longer proposals (around 100 pages)	Costs of PR; PR time	Funding outcome	None	Prospective parallel group study
Holliday and Robotin 2010 [27], Australia	Delphi process for ranking proposals	None	Reviewer agreement	None	Reviewers’ views	Prospective single group study
Mayo et al. 2006 [29], Canada	2-reviewer ‘CLASSIC’ critique method	All-panel members’ independent ‘RANKING’ method	Reviewer agreement; Optimal number of reviewers	Funding outcome	None	Prospective parallel group study
Sattler et al. 2015 [95], USA	11-minute PR training video to improve reviewer reliability	No-training group (included basic video)	Accuracy of rating scale selection; PR time; Reviewer agreement	None	None	Randomised controlled trial
Vo et al. 2015 [100], USA	Virtual PR	Face-to-face PR	Cost per reviewer; PR time	None	Reviewers’ views	Retrospective comparison of several virtual and face-to-face meetings conducted in the same year

**Table 2:** Factors potentially affecting internal validity of the studies, and key uncertainties identified.

Study	Factors supporting internal validity	Factors potentially reducing internal validity	Key uncertainties
Barnett et al. 2015 [23]	Prospective Replicated (4 funding rounds over 2 years)	Single-group cross-sectional type study Descriptive analysis with no quantitative testing Continuous quality improvement assessment but without a defined baseline condition against which to assess changes	Unclear whether data collection instruments were validated Unclear whether applicant views were reported selectively Unclear whether reviewers were aware they were in a research study (unclear performance bias risk)
Fleurence et al. 2014 [57]	Focus groups (providing some stakeholder views) were based on randomly-selected stakeholders	Single-group, cross-sectional/before-after type study	Unclear whether efficiency and effectiveness assessments were prospective or retrospective Unclear whether web survey instrument was validated Unclear timing of focus groups & web survey (unclear recall bias risk) Unclear analysis method for focus group & web survey results Unclear whether reviewers were aware they were in a research study (unclear performance bias risk)
Gallo et al. 2013 [60] Carpenter et al. 2015 [47]	Replicated (2 funding rounds over 2 years) Reviewers unaware they were in a research study	Retrospective Case-control type study	Unclear whether the questionnaire was validated Unclear whether reviewer views were reported selectively Not fully clear who consumer reviewers were Reviewer opinions sought by questionnaire but limited description given
Herbert et al. 2015 [6]	Prospective Parallel 3-group study	Non-randomised The number of reviewers, duration of review, length of proposals & the scoring approaches differed between simplified and standard peer review approaches and therefore effects of these are not separable	Unclear when data on costs and timing were collected in relation to the timing of peer review (unclear recall bias risk) Unclear whether the sample of grant proposals was representative, as it was a convenience sample acquired through existing contacts Unclear whether reviewers were aware they were in a research study (unclear performance bias risk)
Holliday and Robotin 2010 [27]	Prospective	Single-group study Small set of questions may have limited the views that reviewers could provide	Unclear whether data collection instruments were validated Unclear how reviewers’ views were sought and analysed Unclear why views were not reported for all reviewers (unclear risk of selective reporting bias) Timing of data collection unclear (but appears to have been within a four-week period) Unclear whether reviewers were aware they were in a research study (unclear performance bias risk)
Mayo et al. 2006 [29]	Prospective Parallel 2-group study	Non-randomised One member of each two-reviewer group was also a committee member (i.e. non-independence of innovation and comparator) Provision of ranking criteria differed between two-reviewer and committee reviewer groups and therefore effects of these are not separable	Unclear what the 5-point rating scale for proposals was Unclear whether the scoring and ranking sheets were validated Unclear whether reviewers were aware they were in a research study (unclear performance bias risk)
Sattler et al. 2015 [95]	Prospective Randomised Parallel 2-group study Reported that there were no missing data (low attrition bias risk)		Unclear methods of randomisation and whether allocation concealed (risk of selection bias unclear) Unclear whether reviewers were aware they were involved in a research study (blinding not reported; unclear performance bias risk) Unclear how time taken to read grant criteria information was measured Unclear how long after the innovation or comparator the reviewer questionnaire was administered (unclear recall bias risk) Unclear whether the intervention and comparator were run at the same time (unclear contamination bias risk)
Vo et al. 2015 [100]	Replicated (6 parallel innovation sessions within 1 month)	Retrospective Case-control type study No details of the comparator face-to-face meetings reported, so unclear whether they were reflective of usual Agency for Healthcare Research and Quality (AHRQ) face-to-face sessions and how different they were from the innovation	Only limited details of the peer review process reported Unclear how many proposals each reviewer was required to read, how many reviewers were required to read each proposal, or whether this differed between sessions Unclear process for scoring proposals Unclear interval between peer review and questionnaire (unclear recall bias risk) Unclear whether questionnaire was tested or validated Low questionnaire response rate, so unclear representativeness of results Uncertainty around the cost and time savings since similarity of innovation and comparator sessions unclear

**Table 3:** Factors potentially influencing generalisability of the studies.

	Barnett et al. 2015 [23]	Fleurence et al. 2014 [57]	Gallo et al. 2013 2015 [60] [47]	Herbert et al. 2015 [6]	Holliday and Robotin 2010 [27]	Mayo et al. 2006 [29]	Sattler et al. 2015 [95]	Vo et al. 2015 [100]
**Research setting**	Implemented in review sessions of a regional funder AusHSI)	Implemented in review sessions of a national funder (PCORI)	Implemented in review sessions of a national funder (AIBS)	Implemented in review sessions of a national funder (NHMRC)	Implemented in review sessions of a national funder (CCNSW)	Implemented in review session of local university pilot project (MUHCRI)	Study focusing specifically on reliability of scoring in ‘artificial’ experimental setting	Implemented in review sessions of a national funder (AHRQ)
**Topics funded**	Broad range of applied health services research topics (examples reported)	Comparative effectiveness research, but health topics not specified	Broad range of biomedical & health projects (examples reported)	Basic science & public health, but topics not specified	Pancreatic cancer	Broad range including clinical, epi-demiological, health services	Not applicable (experimental study)	Not reported but funder has broad health topic remit
**Award size**	Australian $80,000 per 12-month project	US $1,500,000 in direct costs over 3 years	US $725,000–1,000,000 in direct costs per 3-year project	Not reported	Australian $100,000 per 12-month project	Not reported	Not applicable (experimental study)	Not reported
**Number of proposals**	31 to 89 per review session (4 sessions)	480	1600 over 4 year period (291 to 347 per year)	72 (voluntary sample of submissions)	10	32	Not applicable (experimental study)	198 reviewed (6 to 59 per session), of which 128 discussed (6 to 34 per session) (6 sessions)
**Length of Proposals**	1200 word limit	Not reported	Not reported	9 pages (comparator circa 100 pages)	6 pages	Maximum of 5 pages	Not applicable (experimental study)	Not reported
**Aspects of research proposals assessed**	Applicants’ partnership, research question, method, budget, and expected improvements to health services	8 PCORI Merit criteria (relating to scientific rigour, patient centeredness, engagement of patients and stakeholders)	Scientific merit	Different sections of the full NHMRC proposals form	Scientific merit, innovativeness & level of risk	Innovation: No criteria used. Comparator: research question, background, population characteristics, methods, measures & data analysis (5-point scale).	Not applicable (experimental study)	Not reported
**Number of reviewers**	9 (specialisms reported)	Phase 1: 363 scientists; Phase 2: 111 (59 scientists, 21 patients & 31 stakeholders)	7–12 subject experts + ‘in recent years’ ≥ 1 consumer reviewer per panel	2 (‘journal’ panel), 7 (simplified panel), or 12 (comparator 100)	5	11 (innovation); 2 (comparator)	75 randomly assigned to training and no training; numbers per group not reported	110 (7 to 24 per session)
**Description of reviewers**	Members of AusHSI scientific review committee (specialisms reported)	Scientists, patients & ‘stakeholders’ (caregivers, including nurses & physicians)	Scientists & ‘consumer’ reviewers (had ‘direct experience’ with relevant diseases)	Senior academic researchers (qualification & experience given)	Non-conflicted independent holders of overseas (US) pancreatic cancer grants	Members of a university health centre research institute (committee members and experienced researchers)	Public health professors from research universities across the US	Members of study sections or special emphasis panels (no further details)
**Reviewer recruitment**	Not reported (presumed to follow standard AusHSI process)	Open calls for reviewers & automated search using “Reviewer Finder”	Not reported (presumed to follow standard AIBS process)	Sourced from existing contacts, not selected randomly	Not reported	Selected on content and methodology and statistical expertise(process not specified)	Identified from web-based search for public health programmes	Not reported (presumed to follow standard AHRQ process)
**Reviewer training**	None reported	Training (mandatory) given on PCORI review process in webinars & 1-day face-to-face meeting	Received ‘online and face-to-face ‘orientations’ (when applicable) of the process	None reported	None reported	Reviewers were provided with instructions about the processes (no further details)	The innovation was itself a training programme to improve scoring	30 min of basic training in WebEx software use
**Duration of peer review**	1.5 to 2 months (submission to notification) per funding round (mean review circa 46 min per proposal)	Not reported	Teleconference: mean 19 to 22 min per proposal; face to face: mean 23–29 min per proposal	1.5 days (innovation); 1 week (comparator)	3 Delphi rounds; total 16 days	Not reported	Not applicable (experimental study)	Virtual review: mean 7.2 hours per session (20 min per proposal); comparator 9.8 hours per session; 26 min per proposal
**Review submission tools**	Secure web-based portal	Not reported	Bespoke online system for submitting confidential electronic score sheets	Not reported	Scoring sheet (not described) to submit scores online & funder to collate them	Not reported	Not applicable (experimental study)	WebEx software platform
**Feedback to applicants**	Compre-hensive, from detailed transcription of discussions	None reported	None reported	None reported	None reported	Explanation of comparator (CLASSIC) scores were provided	Not applicable (experimental study)	None reported
**Other issues that might impact on generalisability**	Criteria for AusHSI funding require partnership between healthcare professional and researcher; AusHSI was a new initiative; process included interviews for shortlisted applicants	After this research study PCORI changed their 2-phase peer-review to a 1-phase process	Anonymised written critiques and summary statements were edited by funder’s staff for accuracy and consistency; Ad hoc (i.e. not standing) review panels; about 50% of members were new each year	Authors stated the innovation appeared to attract higher-quality proposals than the standard process			Reviewers did not assess any research proposals; The training innovation focused specifically on the accuracy of interpreting and applying scoring criteria, and did not address all potential areas of training or sources of ‘noise’	Ad hoc (unplanned) peer review sessions

#### Shortening of grant proposals and simplified approaches

**Short proposal with simplified scoring & accelerated peer review (Barnett et al** [23]**)** **Overview**: A streamlined funding protocol for a new health services research stimulus grant awards programme—the Australian Centre for Health Services Innovation (AusHSI). The protocol comprised a short proposal form and accelerated peer review process. The aim was to reduce the content and time required by applicants and reviewers in order to provide rapid and transparent funding decisions.

**Innovation method**: In the protocol applicants are given four weeks to submit electronically a 1,200 word limit form describing the research question, methods, budget and expected impact on health services. Two members of the multi-disciplinary funding committee shortlist proposals and provide written feedback to unsuccessful applicants. Shortlisted applicants attend interviews within 10 days where they make a brief 10 minute presentation to the committee. The proposals are then ranked against a set of criteria and funding is allocated in order of rank until the pre-defined budget limit is met. Successful applicants are notified within two weeks. There is particular emphasis on providing feedback with unsuccessful applicants receiving written feedback and suggested improvements for resubmission.

**Method for assessing the innovation**: The protocol was evaluated as part of a prospective quality improvement evaluation, with internal monitoring data collected at four cross-sectional time points (funding round 1 and 2 in 2012, and round 1 and 2 in 2013). Brief data are also reported on applicants’ views and experiences of the proposal and peer review system.

**Principal results and conclusions**: The average time applicants’ spent preparing their proposals (described as a primary outcome) was seven days over the four funding rounds. The committee members spent on average 36 minutes (range 15–105 minutes) reviewing each proposal prior to the committee meeting where the same reviewers spent 10 minutes discussing each proposal. The mean time from proposal submission to decision notification over the four rounds was seven weeks. Successful research teams were notified within two weeks of interview, which was a maximum of eight weeks after proposal submission. Selected quotations suggest applicants’ views of the protocol were positive. Although for some applicants the 1,200 word limit was challenging the reduction in unnecessary paperwork was appreciated. The feedback given to applicants was also appreciated and they found it enabled them to create better research proposals. In their discussion the authors suggest that, over time, the comprehensive feedback given to applicants who were not successful led to receipt of fewer proposals but of better quality. They conclude that this has improved efficiency for both applicants and reviewers.

**Key strengths and limitations**: The innovation was used in a ‘live’ review round to allocate funding. Overall, limited details are given on the study methods and there is little detailed quantitative or qualitative analysis. The protocol evaluated here was for a relatively smaller scale funding programme, funding award $80,000 (AUSD) for a maximum 12 month project. The findings may not necessarily be applicable to larger funding awards of longer duration.

**Shorter proposal & smaller peer reviewer panel ± face-to-face meeting (Herbert et al** [6]**)** **Overview**: A prospective evaluation of shortened research proposals and simplified peer review processes for the Project Grant scheme of the National Health and Medical Research Council (NHMRC) of Australia. The aim was to identify the agreement between the programme’s official process and two new simplified processes, and the peer review cost savings for the simplified processes.

**Innovation method**: A simplified process where panel members reviewed a nine-page research plan and a two-page track record for each chief investigator. There were two types of simplified panels. One comprised seven members who reviewed proposals during a one and a half day face-to-face meeting (15 minutes discussion of each proposal). The other was a two person ‘journal panel’ (similar to peer review in an academic journal) who independently reviewed and scored proposals (without the two-page track investigator track record). A simplified scoring process was used for both panels (definitely fund, possibly fund, or definitely do not fund). The topics of the proposals were classified as basic science or public health.

**Method for assessing the innovation**: The project was described as a prospective parallel study. The authors compared the outcomes from the two simplified peer review panels in parallel with the existing official NHMRC programme. The study included a sample of 72 research proposals that had been submitted to the official programme and were undergoing assessment in parallel to the research study. The simplified process was initiated by the authors, whilst the official process was independent of the research study (though it was used for purposes of comparison). The official programme comprised 43 panels each with 12 members who meet for a week, and who discuss an average of 91 proposals each of around 100 pages long. Proposals are ranked using a weighted calculation using three criteria-based integer scores (from a one to seven).

**Principal results and conclusions**: The time spent reviewing proposals was similar between the two simplified panels (3.6 to 3.9 hours per proposal on average) (NB. no comparison was made with the official process for this measure). There was near satisfactory agreement in funding decisions between simplified processes and the official processes (72%-74%). The authors estimate that the two simplified panels could result in cost-savings equivalent to AUD $A2.1–$A4.9 million per year compared to the official process (based on costs for the year 2013, equating to a reduction in costs of between 34% to 78%), achieved through reductions in reviewers’ time (and therefore salary costs). The journal panel achieved the highest savings, as no meeting expenses were incurred.

**Key strengths and limitations**: A strength of this study was that the innovation was evaluated in the context of a ‘live’ funding round of a national funder. In terms of limitations there were differences between the official programme and the two simplified processes in terms of how proposals were scored and therefore how funding decisions were made. This may potentially confound the comparison in funding agreement between the processes. The sample of proposals analysed may not be wholly generalisable as they were provided to the study by contacts of the authors, rather than being sampled on a representative basis.

**Peer review panel (11 members) with short proposal vs standard 2-reviewer critique (Mayo et al** [29]**)** **Overview**: A comparison of two methods of peer review on the probability of funding a research proposal: a panel of reviewers who ranked proposals; and a two peer reviewer method. This was a research project funding competition at a major Canadian university medical centre aimed at stimulating pilot clinical research from new investigators and teams. The intention was that they would later submit a full proposal to an external funding agency.

**Innovation method**: A committee of 11 experienced researchers and peer reviewers read and ranked 32 proposals (divided into two streams—new teams and new investigators) and ranked them, without using any explicit criteria (the ‘RANKING’ method). At the start of the committee meeting (before discussion of any results) it was decided that the top two ranked projects in each stream would be funded. For projects ranked three to eight the committee reviewed the ratings from an alternative two-reviewer method (the CLassic Structured Scientific In-depth two reviewer critique ‘CLASSIC’ method) and discussed the projects. Consensus was reached for the next three in each stream to be recommended for funding (thus a total of 10 proposals would be funded).

**Method for assessing the innovation**: The study was a prospective evaluation of two parallel models of peer reviewing. Under the CLASSIC method each proposal was assessed and scored by two assigned peer reviewers using a five point rating scale. The study measured agreement in proposal scoring rank and in the funding decision between the two methods, and the number of reviewers needed to arrive at a consistent ranking.

**Principal results and conclusions**: There was variability in the mean ranks assigned to each proposal between the two methods. The kappa value for agreement in funding decision (based on rank) was 0.36 (95% confidence interval 0.02 to 0.70) indicating poor quality agreement between the two methods. Of the 10 funded projects, the frequency of simulated reviewer pairings drawn from the RANKING committee in which the project failed to meet the funding cut-off ranged from 75% to 9%. Also, projects that were recommended for funding had a 9% to 60% probability of failing to meet the funding cut-off had only two reviewers been assigned (i.e. based on the CLASSIC method). It was estimated that least 10 reviewers would be needed for optimal agreement in funding of proposals. The authors call into question the appropriateness of using the two peer reviewer assessment of research proposals.

**Key strengths and limitations**: The innovation was used in a ‘live’ review round to allocate funding. The study simulated the percentage of possible reviewer pairings (drawn from the 11 member committee) in which a proposal failed to meet the funding cutoff. This was done to mimic the standard practice of (approximate) random allocation of pairs of reviewers to proposals. However, in actuality these proposals were not prospectively distributed amongst pairs of reviewers for review and ranking. Furthermore, ranking criteria differed between groups, confounding comparisons, and the sample of proposals was small.

#### Videoconferencing or teleconferencing approaches

**Teleconference-based peer review meetings (Gallo et al; Carpenter et al** [47, 60]**)** **Overview**: Retrospective comparison of two scientific peer review processes used by the American Institute of Biological Sciences (AIBS) for an anonymous federal funding programme. Specifically, effects on the peer review process and outcomes were compared for face-to-face meetings (held up to 2010) and teleconference meetings (introduced in 2011)[60]. Part of the study focused on examining the effects of discussion on peer review outcomes.[47]

**Innovation method**: Peer reviewers met by teleconference and presented the strengths and weaknesses for each grant proposal using specific review criteria. Each proposals was then discussed by a panel, comprising 7–12 subject matter experts plus one or more ‘consumer’ reviewers, guided by an AIBS chairperson to ensure consistency and fairness. Reviewers then submitted their final scores using an online system. The process was repeated for each proposal, and an overall summary paragraph prepared by assigned reviewers for each proposal, showing the panel’s evaluation and recommendations.

**Method for assessing the innovation**: Case-control type study comparing two years of teleconference peer review meetings (2011–2012) against two years of face-to-face meetings (2009–2010). Face-to-face meetings appear to have had similar structure to teleconferences except that reviewers had to travel to the meeting (usually in a hotel) to participate. Outcomes included: the average time spent discussing each proposal; reviewer agreement estimated using the intra-class correlation coefficient (ICC); the effect on the funding decision of pre-post meeting score changes after discussion (indicated by the proportion of proposals that crossed a theoretical funding threshold); and reviewers’ views on the panel discussions (surveyed at the end of each meeting using a numerical Likert-type scale).

**Principal results and conclusions**: Average review time per proposal was slightly shorter for teleconferences (20.0 minutes) than face-to-face meetings (23.9 minutes) (ANOVA: F_3,61_ = 14.54; p<0.001). Reviewer agreement ranged from ICC = 0.84 to 0.87 across all years, with no clear difference between meeting settings. Slightly more (12.7%) proposals assessed in teleconferences than in face-to-face meetings (10.0%) crossed the funding threshold either way after discussion. After peer review discussion, 19.8% of proposals scored in teleconferences and 15.4% in face-to-face meetings fell within the fundable score range. The authors’ conclusion that most of the outcomes were unaffected by the review setting appears reasonable, although it is unclear how important the reduced discussion time in teleconferences is and unclear whether the reviewers reported any limitations to the process.

**Key strengths and limitations**: The innovation and comparator were used in ‘live’ review rounds of a national funder to allocate funding, with both approaches replicated in two years. Sample size was relatively large (circa 1600 proposals in total; range 291 to 669 per meeting). The retrospective case-control design is a limitation, but reviewer demographic characteristics appear to have been similar across the groups and years. Uncertainties are that the ‘consumer reviewers’ identity is unclear; and only a limited set of reviewers’ views are reported, making it unclear how representative they are.

**WebEx-based virtual peer review meetings (Vo et al** [100]**)** **Overview**: Evaluation of the first six unplanned virtual review sessions conducted during the US 2012 hurricane season at the Agency for Healthcare Research and Quality (AHRQ), to assess their effects on review outcomes and to compare them with five face-to-face peer-review sessions.

**Innovation method**: Virtual online meetings of peer reviewers using WebEx software, which had: audio; high-definition video; real-time content sharing; and the capability to feed up to seven simultaneous webcam videos. A 30-minute basic training session on use of WebEx software was provided. Four Study Section meetings and two Special Emphasis Panel meetings were conducted. In total, 110 reviewers participated, ranging from 7 to 24 per section or panel. Of 194 total grant proposals reviewed, 128 were discussed, ranging from six to 34 proposals per session. Low-scoring proposals were not discussed so as to give reviewers ample time to concentrate on those with higher scores.

**Method for assessing the innovation**: Retrospective case-control type study which compared the six unplanned virtual grant proposal review sessions held in October 2012 against five face-to-face review sessions held in June 2012. The time taken for peer review and the cost of peer review were recorded. Views of reviewers on the advantages and disadvantages of the WebEx software and review process were obtained using a 10-item questionnaire.

**Principal results and conclusions: The** mean time spent discussing each proposal was 20 minutes for virtual review sessions and 26 minutes for face-to-face sessions and the average meeting lengths were 587 minutes and 430 minutes respectively. This gave costs per reviewer per day of US$ 324 and US$1314 respectively (a reduction in costs of 76%). The authors concluded that the virtual review process is a replicable and low cost method of review, but this is subject to the proviso that there are numerous uncertainties around the methods (Table 2). Furthermore, reviewers’ responses to questionnaires indicated that 26% experienced technical difficulties and 33% would not use virtual review again.

**Key strengths and limitations**: The innovation and comparator were used in ‘live’ review rounds of a national funder to allocate funding, with five or six replicate sessions analysed. However, no information about the face-to-face sessions is provided so it is unclear whether these reflected usual AHRQ practice and whether they had comparable proposals, reviewers, and overall processes to the virtual review sessions. There is also uncertainty around several aspects of the virtual peer review process which were not reported, and whether all costs had been accounted for, which limits generalisability.

#### Other approaches

**Modified Delphi process for selecting ‘innovator’ grants (Holliday and Robotin** [27]**)** **Overview**: ‘Modified Delphi’ process, conducted online by the Cancer Council of New South Wales (CCNSW, Australia) for selecting ‘innovator’ grants, based on proposals limited to six pages. The approach was developed because most potential cancer expert peer reviewers were listed as investigators, or had conflicts to declare. This made it inappropriate to use traditional peer review in which local experts are invited as peer reviewers. The grants aimed to support innovative research unlikely to be considered by traditional funding bodies.

**Innovation method**: The process was applied to the 10 best proposals received and involved five non-conflicted experts who held pancreatic cancer research grants in another country (the US). Three Delphi rounds were held over a 16-day period in March 2009 to score: (1) scientific merit (clarity, measurability of the endpoint, scientific quality, originality, adequacy of the study design to achieve the stated goal, whether the potential impact would warrant funding); (2) innovativeness; and (3) level of risk. At the end of each round scores were converted to ranks and the two lowest-ranking proposals at each round were excluded. The four remaining proposals were funded.

**Method for assessing the innovation**: Single-group prospective study in which reviewer agreement was assessed at the end of each round. Reviewers were provided with a table of de-identified scores and an overall ranking of proposals and were asked to advise whether they wished to proceed to the next round, or raise any objections. On completion of the Delphi process feedback was sought from the reviewers on the process, its usefulness, and possible alternatives or modifications (methods for obtaining feedback are not explicitly reported).

**Principal results and conclusions**: The authors’ conclusion was that “the modified Delphi process was an efficient, transparent and equitable method of reviewing novel grant proposals in a specialised field of research, where no local expertise was available” (p. 225). Reviewer feedback indicated that additional discussion would be helpful, suggesting that the innovation may benefit from further modification.

**Key strengths and limitations**: The innovation was used in a ‘live’ review round of a national funder to allocate funding. The process was relatively simple and quick, although it was only tested in one small group of five reviewers, and assessed only 10 proposals. As such, the generalisability is likely to be limited to very small-scale grant programmes or programmes where a subset of the ‘best’ proposals has already been identified for further prioritisation. Further research would be needed to confirm the findings and clarify whether the method could accommodate a larger number of reviewers and proposals. Several aspects of the methodology are unclear, particularly relating to the assessment of reviewer feedback.

**Inclusion of patient-centred stakeholders in peer review meetings (Fleurence et al** [57]**)** **Overview**: The study explored contributions of scientist, patient, and stakeholder reviewers (e.g. nurses, physicians, other caregivers, patient advocates) to the merit-review process of the Patient-Centred Outcomes Research Institute (PCORI) in its inaugural funding round. The rationale was that using scientists alone might bias against novelty, and could lead to selection of proposals similar to the scientists’ interests.

**Innovation method**: The two phase inaugural PCORI merit-review process. In phase one (no discussion), proposals (n = 480) were reviewed by three scientific reviewers who submitted their reviews online. Reviewers received webinar training in PCORI’s review process and criteria. Proposals with average scores in the top third (n = 152) moved to phase two. Proposals in phase two were first given “pre-discussion” scores by two scientists (who did not participate in phase one), one patient and one stakeholder. These four lead reviewers had access to phase one critiques and scores. Patient and stakeholder reviewers based their overall score on three of eight PCORI merit criteria (innovation and potential for improvement; patient centeredness; patient and stakeholder engagement). Proposals in the top two-thirds based on the four lead reviewers’ scores (n = 98) were then given a final “post discussion” score by each member of a 21-person panel (including revised scores from the lead reviewers) during a face-to-face meeting. Lead reviewer scores were available to all reviewers during the discussion. The 25 proposals with the best average post discussion scores were funded. In total 59 scientists, 21 patients and 31 stakeholders participated in phase two.

**Method for assessing the innovation**: Single-group study. Agreement between scientist scores and patient and stakeholder scores was assessed before and after the in-person panel discussions in phase two. The effect on the funding decision of using the 2-phase (scientist, patient and stakeholder) or only a one phase (scientist-only) review process was assessed by comparing proposal rankings after each phase. Web-based surveys and focus groups were used to elicit reviewers’ views.

**Principal results and conclusions**: Of the 25 proposals with the best scores after phase two, only 13 had ranked in the top 25 after phase one, indicating patient and stakeholder reviewers influenced funding decisions. Graphical distributions of scores suggested reviewer agreement improved after discussion for all reviewer types, with strong agreement in post-discussion scores between scientists and non-scientists. Patients and stakeholders appeared to score more critically than scientists. A summary of themes emerging from the surveys and focus groups identified concerns about non-scientists’ technical expertise and a perceived ‘hierarchy’ among reviewers. The authors acknowledge that generalisability of the findings is uncertain.

**Key strengths and limitations**: The innovation was tested in a ‘live’ (inaugural) review round of a national funder, with a relatively large number of proposals, but limited by being a single-group study and unclear whether data collection was prospective or retrospective. Little information is provided about the web survey and focus groups, although it is stated that separate groups were held for a random sample of scientific reviewers, all patients and all stakeholder reviewers.

**Peer reviewer training module to improve scoring accuracy (Sattler et al** [95]**)** **Overview**: Development and evaluation of a brief training programme for grant reviewers that aimed to increase inter-rater reliability, rating scale knowledge, and effort to read National Institutes of Health (NIH) grant review criteria (but did not actually review any proposals).

**Innovation method**: Participants visited a secure website that presented informed consent information, introduced the study, presented an 11-minute training programme video, offered an option to read the criteria for the funding mechanism, and presented a questionnaire. The video emphasized five issues: (1) grant agencies depend on reviewers for accurate information; (2) reviewer scores influence funding decisions; (3) explanation of the NIH rating scale and the definitions of minor, moderate, and major weakness; (4) how to assign evaluation scores that indicate how well the proposal matches the agency’s criteria; and (5) why it is important to carefully read and understand the agency’s criteria. The host stressed that the rating scale used in the video may differ from other grant review rating scales as well as rating scales used in other settings and gave an example of those differences.

**Method for assessing the innovation**: Two-group randomised controlled trial (RCT) comparing training and no-training groups. Participants in the no-training group visited a secure website that presented informed consent information, introduced the study, offered an option to read the criteria for the funding mechanism, and presented a questionnaire. Time to read the grant review criteria was recorded for both groups. Reviewers’ understanding of how to apply scores, and inter-rater agreement in scoring were also assessed for both groups, based on results of the questionnaire. Reviewer agreement was assessed using intra-class correlation coefficients (ICC); Poisson regression was used to assess significance of differences in time to read grant criteria between experienced and novice reviewers.

**Principal results and conclusions**: Inter-rater reliability was significantly higher in the video training group (ICC = 0.89; 95% CI 0.71 to 0.99) than the no-training group (ICC = 0.61; 95% CI 0.32 to 0.96). Participants who received video training spent more time reading grant review criteria (6.1 minutes, SD = 4.8) than those in the no-training group (4.2 minutes, SD = 4.8; Poisson regression, z = 2.17, p = 0.03). Experienced reviewers spent more time reading the criteria (6.0 minutes, SD = 5.6) than novice reviewers (4.2 minutes, SD = 4.0; Poisson regression, z = 3.22, p = 0.001) (reported only for both groups pooled). The authors’ concluded that the training video increased scoring accuracy, inter-rater reliability, and the amount of time reading the review criteria.

**Key strengths and limitations**: The RCT design suggests potentially high internal validity, although superficial reporting means that there are unclear risks of several types of bias. The study has low generalisability due to its focus on a specific part of an NIH scoring system, together with the experimental setting which did not involve assessment of ‘real’ proposals or making any funding decisions.

## Discussion

Our study is the most detailed systematic description of the characteristics of research into the peer review of funding proposals in the health sciences to date. The systematic map has revealed a burgeoning area of investigation, with just under half the studies in the map having been published since 2010. The topics investigated were diverse and the studies were mainly observational in design, typically comprising longitudinal or cross-sectional studies, or retrospective analyses of data collected during funding proposal calls. Experimental studies were very rare, which may demonstrate a preference to study peer review within the context of real world funding programmes, for example on grounds of feasibility, potentially at the expense of internal validity.

Our systematic review included a broad range of innovations and assessed their impact on various measures of effectiveness and efficiency. The majority of the outcomes measured represent ways to make peer review (as well as the research funding process in general) more efficient. The studies showed that innovations could reduce the time spent on peer review and the costs incurred, in varying magnitudes. For example, in one retrospective, case-control-type study, use of teleconferences compared to face-to-face meetings led to a slight reduction in discussion times of up to 10 minutes per proposal, though the overall importance of this reduction was not quantified in terms of changes in costs, or perceived significance. [47, 60] In another retrospective, case-control-type study, use of internet-based video conferences compared to face-to-face meetings resulted in shorter discussion times per proposal (by around six minutes on average) and shorter average meeting lengths (by around 2.5 hours). [100] This was associated with an estimated cost saving of around $1000 (US dollars) per reviewer per day (a 76% reduction), which could be considered an important efficiency improvement. The peer review time per proposal was similar between two variants of an innovation that included shorter proposal forms and smaller peer review panels (3.6 to 3.9 hours), assessed in a prospective parallel group study. [6] The authors of this study estimated that use of these simplified panels could result in cost savings of between $2.1 to $4.9 million (Australian dollars) per year compared to the standard process of a larger panel and a longer proposal form (equating to a reduction in costs of between 34% to 78%). Again, this could represent substantial savings to funders, particularly those that operate at a large scale.

A prospective uncontrolled study [23] which evaluated a simplified process (comprising short proposal forms with accelerated peer review) reported relatively short peer review times per proposal (an average of 36 minutes) and an average time from proposal submission to funding outcome notification of between six to eight weeks. This suggests that accelerated peer review can enable timely funding decisions in certain contexts. The study also provided comprehensive feedback to applicants (both those successful and unsuccessful) on how their proposals could be improved, and the authors noted that over time they received fewer proposals but those submitted were of better quality. However, the trade-off between the costs to funders (in terms of time and resources required to provide detailed feedback to applicants), and the potential benefits to funders and applicants (in terms of production and submission of fewer, better quality, proposals) were not fully quantified by this study. Provision of detailed feedback to applicants has potential to improve the efficiency of the research funding system as a whole, and is an area for future research to investigate.

A number of the studies included in the systematic review measured inter-reviewer agreement, in terms of scores and in funding decisions, with varied findings. For example, good reviewer agreement was found in the study which compared peer review by teleconference discussions with face-to-face meetings, with ICCs ranging between 0.84 and 0.87. [47, 60] The authors suggested that this, and the absence of other differences in review outcomes between the two approaches, supports the case for moving to teleconferences. In contrast, a study which compared ranking of proposals by a committee of 11 reviewers against ranking of proposals by two peer reviewers found poor reviewer agreement in ranking scores (and therefore decisions to fund) as measured by a kappa score of 0.36. [29] Lack of good agreement might not necessarily be a limitation of peer review if this is offset by other efficiency benefits such as time and cost reductions. However, none of the studies included in our systematic review measured all of these outcomes, so possible trade-offs among different aspects of efficiency cannot be ascertained currently.

There were mixed findings across the studies indicating perceived benefits but also drawbacks of the innovations. For example, in the study in which patients and care-giving stakeholders peer reviewed funding proposals alongside scientific reviewers, scientists appreciated the perspectives offered by patients and stakeholders and there was recognition of a collegial and respectful process. [57] However, there was concern from scientists about the level of technical expertise of some non-scientist reviewers. The study comparing internet-based video conferences to face-to-face meetings [100] reported both positive and negative views expressed by peer reviewers. Perceived advantages included less travel, decreased costs, and faster reviews. However, some technical problems were experienced, and there was concern that video-conferences might impair interaction among reviewers and result in less thorough reviews. It is important that any implementation of these peer review innovations takes into account the limitations, and future evaluations should thoroughly evaluate process issues to facilitate optimal planning and execution of peer review activity.

Our findings can be contextualised with those of a non-systematic literature review by Guthrie et al.[105] published in 2017 which included 105 empirical articles on the effectiveness and burden of peer review for grant funding. That review had a broader focus than our systematic review, covering issues such as bias and fairness, reliability, timeliness of peer review, and the burden of peer review on the research system as a whole. It also included studies of peer review in disciplines other than health sciences. The review included many of the studies included in our systematic review, but described them in less detail. Notably, Guthrie et al.’s review incorporated a different conceptualisation of effectiveness and efficiency than in our review: ‘effectiveness’ is a multi-dimensional concept that incorporates factors such as whether peer review selects the ‘best’ research; whether it is reliable, fair, accountable, timely and has the confidence of key stakeholders. The ‘burden’ of peer review on the research system is a concept that incorporates the time, resources and costs expended in the production and review of grant applications. ‘Efficiency’ is the trade-off between effectiveness and burden. Thus, an efficient peer review system is one that has one or more markers of effectiveness whilst being low in system burden. Guthrie et al. [105] found there was a lack of evidence about the overall efficiency of peer review of grant applications. In terms of markers of effectiveness they found evidence to indicate a bias against innovative research, and evidence of the poor prediction of peer review on future research performance. They found some evidence to suggest a high burden on applicants, though much of the research evidence in their review has focused on reducing burden on funders and reviewers. Applying Guthrie’s conceptualisation to our systematic review results there is evidence to show a reduction in burden for funders (which we refer to as efficiency in our review). However, evidence for the effectiveness of peer review in our systematic review is limited to whether innovations which aim to reduce peer review burden can lead to the same research applications being funded as would have been funded under existing (more burdensome) peer review systems. The studies in our systematic review did not assess other markers of effectiveness such as the predictive ability to identify the best research. Thus, we cannot conclude that there is strong evidence to support improving the ‘efficiency’ (as defined by Guthrie et al,[105]) of peer review of grant applications, but we can conclude there is evidence (albeit with methodological limitations) on burden reduction.

Our research used systematic methods to identify, collate, appraise and analyse the evidence, employing standard approaches in evidence synthesis. [106, 107] Extensive internet searching was conducted to identify material not formally published in academic journals. Quality assurance procedures, such as independent screening and data checking, were used where possible to minimise bias and error. However, there were some potential limitations of this study. We could not check the reference lists of all studies included in the map to identify any additional relevant studies, though we did check the reference lists of all studies included in the systematic review. Not all of the keywords applied to studies included in the map were checked by a second reviewer. However, as mentioned above, following checking of a random sample of studies the level of reliability between reviewers was considered sufficient as few amendments were necessary. We restricted inclusion to studies published in the English language. It is unknown whether there is a significant pool of relevant evidence published in other languages. The scope of our evidence synthesis is limited to studies of peer review of research proposals in health; we did not investigate studies of peer review of research proposals in other disciplines. Whilst it is possible that findings from studies in non-health disciplines could also have relevance to health research, a substantial effort would be required to synthesise the evidence across multiple disciplines. Our findings suggest, however, that even within health research the studies had limited generalisability.

A strength of this evidence synthesis was the close consultation with stakeholders throughout the project, and in particular their role in setting the focus for the systematic review. [21] It should be reiterated that the scope of the systematic review was to focus on peer review innovations evaluated for effectiveness and efficiency. Only a small proportion (around 10%) of the evidence from the map met the inclusion criteria for the review, meaning that there remains a larger pool of evidence that could be included in future systematic reviews focusing on other aspects of peer review. Also of note, our systematic review included studies of innovations, which we defined as being new activity distinct from existing practice (or in addition to existing practice). Some of the literature evaluated only what appeared to be existing peer review practice, and useful information could be gleaned from these studies in further reviews.

## Conclusions

This project has found that there is increasing international research activity into the peer review of health research funding. Overall, it appears that simplifying peer review by shortening proposals, using smaller panels of reviewers and accelerating the process could reduce the time needed for review, speed up the general process, and reduce costs. However, this might come at the expense of peer review quality, a key aspect that has not been fully assessed. Virtual peer review using videoconferencing or teleconferencing appears promising for reducing costs by avoiding the need for reviewers to travel, but again any consequences for the quality of the peer review itself have not been adequately assessed. All of the eight studies included in the systematic review were relatively weak methodologically or had variable generalisability, which limits how much emphasis should be placed on their results.

Given the methodological limitations of the evidence included in this systematic review it is not possible to recommend direct implementation of these innovations currently. However, many of them appear promising based on current evidence and could be adapted as necessary by funders and subjected to evaluation. Future evaluations should be conducted to a sufficient standard, to ensure high internal and external validity. In particular, we have identified a number of measures of generalisability of studies which we recommend that evaluators incorporate into the design and reporting of their work (Table 3). Where feasible, experimental evaluations, including RCTs, should be conducted including economic evaluation to assess costs of peer review innovations as this is lacking in the currently available evidence.

## Supporting information

S1 Appendix Medline search strategy. (DOCX)

S2 Appendix Further detail on stakeholder topic prioritisation. (DOCX)

S3 Appendix PRISMA checklist. (DOC)

S1 Database Systematic map database. (XLSX)

S1 Protocol Protocol. (PDF)

S1 Table Supporting results tables. (DOCX)

## References

[1] Godlee F, Jefferson T. Peer Review in Health Sciences, 2nd Edition London: BMJ Books; 2003.

[2] Gluckman P. Which science to fund: time to review peer review? Auckland: Office of the Prime Minister’s Science Advisory Committee (New Zealand); 2012.

[3] Schroter S, Groves T, Hojgaard L. Surveys of current status in biomedical science grant review: funding organisations’ and grant reviewers’ perspectives. BMC Med. 2010;8:62 doi: 10.1186/1741-7015-8-62 20961441 10.1186/1741-7015-8-62 PMC2974654

[4] Guthrie S, Guérin B, Wu H, Ismail S, Wooding S. Alternatives to Peer Review in Research Project Funding. Cambridge: RAND Europe; 2013.

[5] Lee CJ, Sugimoto CR, Zhang G, Cronin B. Bias in peer review. Journal of the American Society for Information Science and Technology. 2013;64(1):2–17.

[6] Herbert DL, Graves N, Clarke P, Barnett AG. Using simplified peer review processes to fund research: a prospective study. BMJ Open. 2015;5(7):e008380 doi: 10.1136/bmjopen-2015-008380 26137884 10.1136/bmjopen-2015-008380 PMC4499682

[7] Barnett A, Graves N, Clarke P, Herbert D. The impact of a streamlined funding application process on application time: two cross-sectional surveys of Australian researchers. BMJ Open. 2015;5(e006912):1–6. 10.1136/bmjopen-2014-006912 PMC4298094 25596201

[8] Boden M. Peer review: a report to the Advisory Board for the Research Councils from the Working Goup on peer review. London; 1990.

[9] Graves N, Barnett AG, Clarke P. Funding grant proposals for scientific research: retrospective analysis of scores by members of grant review panel. Br Med J. 2011;343:d4797. 21951756 10.1136/bmj.d4797 PMC3181233

[10] Li D, Agha L. Research funding. Big names or big ideas: do peer-review panels select the best science proposals? Science. 2015;348(6233):434–8. 25908820 10.1126/science.aaa0185

[11] Powell K. Research funding: Making the cut. Nature. 2010;467(7314):383–5. doi: 10.1038/467383a 20864969 10.1038/467383a

[12] Wessely S, Wood FQ. Chapter 2: Peer review of grant applications: a systematic review In: Godlee F, Jefferson T, editors. Peer review in health sciences. London BMJ Books; 1999 p. 14–31.

[13] Lamont M. How Professors Think Inside the Curious World of Academic Judgement. Cambridge, Massachusetts: Harvard University Press; 2009.

[14] Ismail S, Farrands A, Wooding S. Evaluating Grant Peer Review in the Health Sciences. Santa Monica: RAND Corporation; 2009.

[15] Demicheli V, Di Pietrantonj C. Peer review for improving the quality of grant applications. The Cochrane database of systematic reviews. 2007(2):MR000003. 10.1002/14651858.MR000003.pub2 PMC8973940 17443627

[16] Frampton GK, Harris P, Cooper K, Cooper T, Cleland J, Jones J, et al Educational interventions for preventing vascular catheter bloodstream infections in critical care: evidence map, systematic review and economic evaluation. Health technology assessment (Winchester, England). 2014;18(15):1–365. 10.3310/hta18150 PMC4781186 24602781

[17] Miake-Lye IM, Hempel S, Shanman R, Shekelle PG. What is an evidence map? A systematic review of published evidence maps and their definitions, methods, and products. Syst Rev. 2016;5(1):1–21. 26864942 10.1186/s13643-016-0204-x PMC4750281

[18] Schucan Bird K, Newman M, Hargreaves K, Sawtell M. Workplace-based learning for undergraduate and pre-registration healthcare professionals: A systematic map of the UK research literature 2003–2013. London: EPPI-Centre, Social Science Research Unit, UCL Institute of Education, University College London.; 2015.

[19] Shepherd J, Kavanagh J, Picot J, Cooper K, Harden A, Barnett-Page E, et al The effectiveness and cost-effectiveness of behavioural interventions for the prevention of sexually transmitted infections in young people aged 13–19: a systematic review and economic evaluation. Health technology assessment (Winchester, England). 2010;14(7):1–206, iii-iv. 10.3310/hta14070 20178696

[20] Wang DD, Shams-White M, Bright OJ, Parrott JS, Chung M. Creating a literature database of low-calorie sweeteners and health studies: evidence mapping. BMC medical research methodology. 2016;16:1 doi: 10.1186/s12874-015-0105-z 26728979 10.1186/s12874-015-0105-z PMC4700619

[21] Rees R, Oliver S. Stakeholder perspectives and participation in systematic reviews In: Gough D, Oliver S, Thomas J, editors. An Introduction to Systematic Reviews. London: Sage; 2012 p. 17–34.

[22] Abdoul H, Perrey C, Amiel P, Tubach F, Gottot S, Durand-Zaleski I, et al Peer review of grant applications: criteria used and qualitative study of reviewer practices. PLoS ONE. 2012;7(9):e46054 doi: 10.1371/journal.pone.0046054 23029386 10.1371/journal.pone.0046054 PMC3460995

[23] Barnett AG, Herbert DL, Campbell M, Daly N, Roberts JA, Mudge A, et al Streamlined research funding using short proposals and accelerated peer review: an observational study. BMC Health Serv Res. 2015;15:55 doi: 10.1186/s12913-015-0721-7 25888975 10.1186/s12913-015-0721-7 PMC4324047

[24] Fogelholm M, Leppinen S, Auvinen A, Raitanen J, Nuutinen A, Vaananen K. Panel discussion does not improve reliability of peer review for medical research grant proposals. J Clin Epidemiol. 2012;65(1):47–52. doi: 10.1016/j.jclinepi.2011.05.001 21831594 10.1016/j.jclinepi.2011.05.001

[25] Grant J, Low L. Women and peer review An audit of the Wellcome Trust’s decision-making on grants London, UK: Wellcome Trust. Unit for Policy Research in Science and Medicine (PRISM); 1997.

[26] Green JG, Calhoun F, Nierzwicki L, Brackett J, Meier P. Rating intervals: an experiment in peer review. Faseb J. 1989;3(8):1987–92. 2721858 10.1096/fasebj.3.8.2721858

[27] Holliday C, Robotin M. The Delphi process: a solution for reviewing novel grant applications. Int J Gen Med. 2010;3:225–30. 20830198 10.2147/ijgm.s11117 PMC2934605

[28] Lindner MD, Nakamura RK. Examining the Predictive Validity of NIH Peer Review Scores.[Erratum appears in PLoS One. 2015;10(6):e0132202; PMID: 26121031]. PLoS ONE. 2015;10(6):e0126938 doi: 10.1371/journal.pone.0126938 26039440 10.1371/journal.pone.0126938 PMC4454673

[29] Mayo NE, Brophy J, Goldberg MS, Klein MB, Miller S, Platt RW, et al Peering at peer review revealed high degree of chance associated with funding of grant applications. J Clin Epidemiol. 2006;59(8):842–8. doi: 10.1016/j.jclinepi.2005.12.007 16828678 10.1016/j.jclinepi.2005.12.007

[30] Mow KE. Research Grant Funding and Peer Review in Australian Research Councils. PhD thesis. Canberra: University of Canberra. Administrative Studies; 2009.

[31] Pina DG, Hren D, Marusic A. Peer Review Evaluation Process of Marie Curie Actions under EU's Seventh Framework Programme for Research. PLoS ONE. 2015;10(6):e0130753 doi: 10.1371/journal.pone.0130753 26126111 10.1371/journal.pone.0130753 PMC4488366

[32] Street J, Baum F, Anderson IP. Is peer review useful in assessing research proposals in Indigenous health? A case study. Health Res Policy Syst. 2009;7:2 doi: 10.1186/1478-4505-7-2 19216770 10.1186/1478-4505-7-2 PMC2654449

[33] Higgins JPT, Altman DG, Gøtzsche PC, Jüni P, Moher D, Oxman AD, et al The Cochrane Collaboration’s tool for assessing risk of bias in randomised trials. Br Med J. 2011;343. 10.1136/bmj.d5928 PMC3196245 22008217

[34] AMRC (Association of Medical Research Charities). A house in good order: a report on the AMRC peer review audit 2011. London, UK: AMRC; 2012.

[35] Andejeski Y, Bisceglio IT, Dickersin K, Johnson JE, Robinson SI, Smith HS, et al Quantitative impact of including consumers in the scientific review of breast cancer research proposals. J Womens Health Gend Based Med. 2002;11(4):379–88. doi: 10.1089/152460902317586010 12150500 10.1089/152460902317586010

[36] Berg J. NIGMS Feedback Loop Blog [Internet]. USA: National Institute of General Medical Sciences. 2011. [cited 2016]. https://loop.nigms.nih.gov/category/peer-review/page/3/.

[37] Bielski A, Harris R, Gillis N. Summary report of comments received on NIH system to support biomedical and behavioral research and peer review. Bethesda, MD, USA: Ripple Effect Communications, Inc.; 2007.

[38] Bornmann L, Daniel HD. Criteria used by a peer review committee for selection of research fellows—A boolean probit analysis. Int J Sel Assess. 2005;13(4):296–303.

[39] Bornmann L, Daniel HD. Selection of research fellowship recipients by committee peer review. Reliability, fairness and predictive validity of Board of Trustees’ decisions. Scientometrics. 2005;63(2):297–320.

[40] Bornmann L, Daniel HD. Potential sources of bias in research fellowship assessments: effects of university prestige and field of study. Res Evaluat. 2006;15(3):209–19.

[41] Bornmann L, Daniel HD. Selecting scientific excellence through committee peer review—A citation analysis of publications previously published to approval or rejection of post-doctoral research fellowship applicants. Scientometrics. 2006;68(3):427–40.

[42] Bornmann L, Mutz R, Daniel HD. Row-column (RC) association model applied to grant peer review. Scientometrics. 2007;73(2):139–47.

[43] Bornmann L, Mutz R, Daniel HD. Latent Markov modeling applied to grant peer review. J Informetr. 2008;2(3):217–28.

[44] Boyack KW, Chen MC, Chacko G. Characterization of the peer review network at the Center for Scientific Review, National Institutes of Health. PLoS ONE. 2014;9(8):e104244 doi: 10.1371/journal.pone.0104244 25119140 10.1371/journal.pone.0104244 PMC4132088

[45] Cabezas-Clavijo A, Robinson-Garcia N, Escabias M, Jimenez-Contreras E. Reviewers’ ratings and bibliometric indicators: hand in hand when assessing over research proposals? PLoS ONE. 2013;8(6):e68258 doi: 10.1371/journal.pone.0068258 23840840 10.1371/journal.pone.0068258 PMC3695904

[46] Campbell D, Picard-Aitken M, Cote G, Caruso J, Valentim R, Edmonds S, et al Bibliometrics as a Performance Measurement Tool for Research Evaluation: The Case of Research Funded by the National Cancer Institute of Canada. Am J Eval. 2010;31(1):66–83.

[47] Carpenter AS, Sullivan JH, Deshmukh A, Glisson SR, Gallo SA. A retrospective analysis of the effect of discussion in teleconference and face-to-face scientific peer-review panels. BMJ Open. 2015;5(9):e009138 doi: 10.1136/bmjopen-2015-009138 26351194 10.1136/bmjopen-2015-009138 PMC4563222

[48] Carter G. Peer review, citations, and biomedical research policy: NIH grants to medical school faculty. Santa Monica, CA, USA: RAND; 1974.

[49] Carter G. A citation study of the NIH peer review process. Santa Monica, CA, USA: RAND; 1978.

[50] Carter G. What we know and do not know about the NIH peer review system. Santa Monica, CA, USA; 1982.

[51] Clarke P, Herbert D, Graves N, Barnett AG. A randomized trial of fellowships for early career researchers finds a high reliability in funding decisions. J Clin Epidemiol. 2016;69:147–51. doi: 10.1016/j.jclinepi.2015.04.010 26004515 10.1016/j.jclinepi.2015.04.010

[52] Claveria LE, Guallar E, Cami J, Conde J, Pastor R, Ricoy JR, et al Does peer review predict the performance of research projects in health sciences? Scientometrics. 2000;47(1):11–23.

[53] Das NK, Froehlich LA. Quantitative evaluation of peer review of program project and center applications in allergy and immunology. J Clin Immunol. 1985;5(4):220–7. 4044784 10.1007/BF00929456

[54] Doyle JM, Quinn K, Bodenstein YA, Wu CO, Danthi N, Lauer MS. Association of percentile ranking with citation impact and productivity in a large cohort of de novo NIMH-funded R01 grants. Molecular Psychiatry. 2015;20(9):1030–6. doi: 10.1038/mp.2015.71 26033238 10.1038/mp.2015.71 PMC5526589

[55] DTZ Consulting & Research (for RCUK). Analysis of the external costs of peer review. Swindon, UK: DTZ Consulting & Research; 2006.

[56] Fang FC, Bowen A, Casadevall A. NIH peer review percentile scores are poorly predictive of grant productivity. elife. 2016;5. 10.7554/eLife.13323 PMC4769156 26880623

[57] Fleurence RL, Forsythe LP, Lauer M, Rotter J, Ioannidis JP, Beal A, et al Engaging patients and stakeholders in research proposal review: the patient-centered outcomes research institute. Ann Intern Med. 2014;161(2):122–30. doi: 10.7326/M13-2412 25023251 10.7326/M13-2412

[58] Fonseca L, Rangel V, Lustosa P, Lannes D, Aguiar LC, Flavoni L, et al Productivity versus promised results: one of the dilemmas of biotechnology in Brazil. Braz J Med Biol Res. 1994;27(12):2709–20. 7549995

[59] Fuhrer MJ, Grabois M. Grant application and review procedures of the National Institute of Handicapped Research: survey of applicant and peer reviewer opinions. Arch Phys Med Rehabil. 1985;66(5):318–21. 3159374

[60] Gallo SA, Carpenter AS, Glisson SR. Teleconference versus face-to-face scientific peer review of grant application: effects on review outcomes. PLoS ONE. 2013;8(8):e71693 doi: 10.1371/journal.pone.0071693 23951223 10.1371/journal.pone.0071693 PMC3740535

[61] Gallo SA, Carpenter AS, Irwin D, McPartland CD, Travis J, Reynders S, et al The validation of peer review through research impact measures and the implications for funding strategies. PLoS ONE. 2014;9(9):e106474 doi: 10.1371/journal.pone.0106474 25184367 10.1371/journal.pone.0106474 PMC4153641

[62] Gallo SA, Lemaster M, Glisson SR. Frequency and Type of Conflicts of Interest in the Peer Review of Basic Biomedical Research Funding Applications: Self-Reporting Versus Manual Detection. Sci Eng Ethics. 2016;22(1):189–97. doi: 10.1007/s11948-015-9631-7 25649072 10.1007/s11948-015-9631-7

[63] Gilkey MB. Consumer advocates in the peer review of cancer-related research: Experience, representation, and the lived body. PhD thesis. Baltimore, Maryland: Johns Hopkins University; 2012.

[64] Gilkey MB. Supporting cancer survivors’ participation in peer review: perspectives from NCI's CARRA program. J Cancer Surviv. 2014;8(1):114–20. doi: 10.1007/s11764-013-0318-2 24214497 10.1007/s11764-013-0318-2 PMC3945408

[65] Glantz SA, Bero LA. Inappropriate and appropriate selection of ‘peers’ in grant review. Jama. 1994;272(2):114–6. 8015118

[66] Grace WC, Levitin T, Cole S. Characteristics of successfully recruited grant application peer reviewers [abstract]. 4th International Congress on Peer Review in Biomedical Publication, September 14–16, 2001 in Barcelona, Spain [Internet]. 2001. http://onlinelibrary.wiley.com/o/cochrane/clcmr/articles/CMR-4040/frame.html.

[67] Hodgson C. Evaluation of cardiovascular grant-in-aid applications by peer review: influence of internal and external reviewers and committees. Can J Cardiol. 1995;11(10):864–8. 7489524

[68] Hodgson C. How reliable is peer review? An examination of operating grant proposals simultaneously submitted to two similar peer review systems. J Clin Epidemiol. 1997;50(11):1189–95. 9393374 10.1016/s0895-4356(97)00167-4

[69] Hume KM, Giladi AM, Chung KC. Factors impacting successfully competing for research funding: an analysis of applications submitted to the Plastic Surgery Foundation. Plast Reconstr Surg. 2015;135(2):429e–35e. doi: 10.1097/PRS.0000000000000904 25626827 10.1097/PRS.0000000000000904 PMC4809526

[70] Johnson V. Statistical analysis of the National Institutes of Health peer review system. Proc Natl Acad Sci USA. 2008;105(32):11076–80. doi: 10.1073/pnas.0804538105 18663221 10.1073/pnas.0804538105 PMC2488382

[71] Kaatz A, Magua W, Zimmerman DR, Carnes M. A quantitative linguistic analysis of National Institutes of Health R01 application critiques from investigators at one institution. Acad Med. 2015;90(1):69–75. doi: 10.1097/ACM.0000000000000442 25140529 10.1097/ACM.0000000000000442 PMC4280285

[72] Kaatz AC. Quantitative text analysis of R01 grant reviews from the National Institutes of Health (NIH). University of Wisconsin—Madison: University of Wisconsin—Madison; 2013.

[73] Kalberer JT Jr. Treatment of young investigators in the National Cancer Program. J Natl Cancer Inst. 1979;63(4):1097–103. 480383

[74] Kaltman JR, Evans FJ, Danthi NS, Wu CO, DiMichele DM, Lauer MS. Prior publication productivity, grant percentile ranking, and topic-normalized citation impact of NHLBI cardiovascular R01 grants. Circ Res. 2014;115(7):617–24. doi: 10.1161/CIRCRESAHA.115.304766 25214575 10.1161/CIRCRESAHA.115.304766 PMC4163934

[75] Kotchen TA, Lindquist T, Malik K, Ehrenfeld E. NIH peer review of grant applications for clinical research. Jama. 2004;291(7):836–43. doi: 10.1001/jama.291.7.836 14970062 10.1001/jama.291.7.836

[76] Kotchen TA, Lindquist T, Miller Sostek A, Hoffmann R, Malik K, Stanfield B. Outcomes of National Institutes of Health peer review of clinical grant applications. J Investig Med. 2006;54(1):13–9. 16409886 10.2310/6650.2005.05026

[77] Langfeldt L. The decision-making constraints and processes of grant peer review, and their effects on the review outcome. Soc Stud Sci. 2001;31(6):820–41.

[78] Lauer MS, Danthi NS, Kaltman J, Wu C. Predicting Productivity Returns on Investment: Thirty Years of Peer Review, Grant Funding, and Publication of Highly Cited Papers at the National Heart, Lung, and Blood Institute. Circ Res. 2015;117(3):239–43. doi: 10.1161/CIRCRESAHA.115.306830 26089369 10.1161/CIRCRESAHA.115.306830 PMC4506707

[79] Lobb R, Petermann L, Manafo E, Keen D, Kerner J. Networking and knowledge exchange to promote the formation of transdisciplinary coalitions and levels of agreement among transdisciplinary peer reviewers. J Public Health Manag Pract. 2013;19(1):E9–20. doi: 10.1097/PHH.0b013e31823991c2 22990496 10.1097/PHH.0b013e31823991c2

[80] Martin MR, Kopstein A, Janice JM. An analysis of preliminary and post-discussion priority scores for grant applications peer reviewed by the Center for Scientific Review at the NIH. PLoS ONE. 2010;5(11):e13526 doi: 10.1371/journal.pone.0013526 21103331 10.1371/journal.pone.0013526 PMC2984433

[81] Martin MR, Lindquist T, Kotchen TA. Why are peer review outcomes less favorable for clinical science than for basic science grant applications? Am J Med. 2008;121(7):637–41. doi: 10.1016/j.amjmed.2008.03.031 18589061 10.1016/j.amjmed.2008.03.031 PMC2517849

[82] Monahan A, Stewart D. The role of lay panelists on grant review panels. Chronic Dis Can. 2003;24(2–3):70–4. 12959677

[83] Mutz R, Bornmann L, Daniel HD. Heterogeneity of inter-rater reliabilities of grant peer reviews and its determinants: a general estimating equations approach. PLoS ONE. 2012;7(10):e48509 doi: 10.1371/journal.pone.0048509 23119041 10.1371/journal.pone.0048509 PMC3485362

[84] NIH (National Institutes of Health). Enhancing peer review. Survey results report (2010). Bethesda, MD, USA: National Institutes of Health; 2010.

[85] NIH (National Institutes of Health). Enhancing peer review. Survey results report (2013). Bethesda, MD, USA: National Institutes of Health; 2013.

[86] Olsson CA, Kennedy WA 2nd. Urology peer review at the National Institutes of Health. J Urol. 1995;154(5):1866–9. 7563369

[87] Quaglio G, Guardabasso V, Olesen OF, Draghia-Akli R. The selection of experts evaluating health projects for the EU Sixth Framework Program. J Public Health. 2011;19(5):445–52. doi: 10.1007/s10389-011-0395-5 21957333 10.1007/s10389-011-0395-5 PMC3172421

[88] Ramos MA, Fox A, Simon EP, Horowitz CR. A community-academic partnership to address racial/ethnic health disparities through grant-making. Public Health Rep. 2013;128 Suppl 3:61–7. 24179281 10.1177/00333549131286S310 PMC3945451

[89] Rangel SJ, Efron B, Moss RL. Recent trends in National Institutes of Health funding of surgical research. Ann Surg. 2002;236(3):277–86; discussion 86–7. 12192314 10.1097/00000658-200209000-00004 PMC1422581

[90] RCUK (Research Councils UK). Report of the Research Councils UK Efficiency and Effectiveness of Peer Review project. Swindon, UK: RCUK; 2006.

[91] RCUK (Research Councils UK). RCUK Response to the project report & consultation on the efficiency and effectiveness of peer review. Swindon, UK: RCUK; 2007.

[92] RCUK (Research Councils UK). Summary of the analysis of the responses received to the RCUK efficiency and effectiveness of peer review consultation2007. http://www.rcuk.ac.uk/documents/documents/analysisresponsepeer-pdf/.

[93] Reinhart M. Peer review of grant applications in biology and medicine. Reliability, fairness, and validity. Scientometrics. 2009;81(3):789–809.

[94] Russell AS, Thorn BD, Grace M. Peer review: a simplified approach. J Rheumatol. 1983;10(3):479–81. 6887172

[95] Sattler DN, McKnight PE, Naney L, Mathis R. Grant Peer Review: Improving Inter-Rater Reliability with Training. PLoS ONE. 2015;10(6):e0130450 doi: 10.1371/journal.pone.0130450 26075884 10.1371/journal.pone.0130450 PMC4468126

[96] Snell RR. Menage a quoi? Optimal number of peer reviewers. PLoS ONE. 2015;10(4):e0120838 doi: 10.1371/journal.pone.0120838 25830238 10.1371/journal.pone.0120838 PMC4382286

[97] Street J, Baum F, Anderson I. Developing a collaborative research system for Aboriginal health. Aust N Z J Public Health. 2007;31(4):372–8. 17725020 10.1111/j.1753-6405.2007.00090.x

[98] Taylor M. Of molecules, mice, and men: The relationship of biological complexity of research model to final rating in the grant peer review process of the Heart and Stroke Foundation of Canada [abstract]. 4th International Congress on Peer Review in Biomedical Publication, September 14–16, 2001 in Barcelona, Spain [Internet]. 2001. http://onlinelibrary.wiley.com/o/cochrane/clcmr/articles/CMR-4073/frame.html.

[99] Vener KJ, Feuer EJ, Gorelic L. A statistical model validating triage for the peer review process: keeping the competitive applications in the review pipeline. Faseb J. 1993;7(14):1312–9. 8224604 10.1096/fasebj.7.14.8224604

[100] Vo NM, Trocki R. Virtual and Peer Reviews of Grant Applications at the Agency for Healthcare Research and Quality. South Med J. 2015;108(10):622–6. 26437196 10.14423/SMJ.0000000000000353

[101] Wang Q, Sandstrom U. Defining the role of cognitive distance in the peer review process with an explorative study of a grant scheme in infection biology. Res Evaluat. 2015;24(3):271–81.

[102] Whaley AL. An objective rating form to evaluate grant proposals to the Hogg Foundation for Mental Health: a pilot study of implementation. Eval Rev. 2006;30(6):803–16. doi: 10.1177/0193841X06288737 17093109 10.1177/0193841X06288737

[103] Wiener SL, Urivetzky M, Bregman D, Cohen J, Eich R, Gootman N, et al Peer review: inter-reviewer agreement during evaluation of research grant applications. Clin Res. 1977;25(5):306–11. 10304719

[104] Wisely J, Haines A. Commissioning a national programme of research and development on the interface between primary and secondary care. Br Med J. 1995;311(7012):1080–2. 7580670 10.1136/bmj.311.7012.1080 PMC2551375

[105] Guthrie S, Ghiga I, Wooding S. What do we know about grant peer review in the health sciences? [version 1; referees: 1 approved, 1 approved with reservations]2017. 10.12688/f1000research.11917.1 PMC5883382 29707193

[106] Higgins JP, Green S. Cochrane Handbook for Systematic Reviews of Interventions Version 5.1.0 [updated March 2011]. The Cochrane Collaboration; 2011.

[107] Moher D, Liberati A, Tetzlaff J, Altman DG, The PG. Preferred Reporting Items for Systematic Reviews and Meta-Analyses: The PRISMA Statement. PLoS Medicine. 2009;6(7):e1000097 doi: 10.1371/journal.pmed.1000097 19621072 10.1371/journal.pmed.1000097 PMC2707599

