# Supplementary material for: Peer review of health research funding proposals: A systematic map and systematic review of innovations for effectiveness and efficiency
Source: PLoS One. 2018 May 11;13(5):e0196914. doi: 10.1371/journal.pone.0196914 (PMC5947897; doi:10.1371/journal.pone.0196914)
Supplement: S1 Appendix — (DOCX) [file pone.0196914.s001.docx]

**S1 Appendix – Medline search strategy**

This strategy was adapted for use in other databases as necessary

1 "peer review"/ or peer review, research/ (12076)

2 (peer adj review*).tw. (18395)

3 1 or 2 (26600)

4 (grant* adj2 (financ* or budget* or allocat*)).tw. (151)

5 (research adj2 (fund* or grant* or proposal* or application* or applicant* or

submission* or budget* or financ*)).tw. (14327)

6 (program* adj grant*).tw. (128)

7 (grant adj2 (application* or applicant* or submission*)).tw. (511)

8 (grant adj2 proposal*).tw. (257)

9 (grant adj2 award*).tw. (201)

10 Financing, Organized/ or Financing, Government/ (25154)

11 Research Support as Topic/ec (2539)

12 (fund* and decision*).tw. (9348)

13 (grant* and decision*).tw. (1241)

14 (protocol* adj5 (grant* or fund*)).tw. (352)

15 Research Support as Topic/ec (2539)

16 Financial Management/ (15951)

17 or/4‐16 (65714)

18 3 and 17 (1106)

19 ("peer review*" and grant*).ti. (65)

20 ("peer review*" and fund*).ti. (35)

21 18 or 19 or 20 (1120)

22 ("peer review" and process* and research and grant*).tw. (67)

23 ("peer review" and process* and research and fund*).tw. (125)

24 21 or 22 or 23 (1173)

25 remove duplicates from 24 (1160)

26 from 25 keep 1‐1000 (1000)

27 from 25 keep 1001‐1160 (160)
