# Supplementary material for: Peer review of health research funding proposals: A systematic map and systematic review of innovations for effectiveness and efficiency
Source: PLoS One. 2018 May 11;13(5):e0196914. doi: 10.1371/journal.pone.0196914 (PMC5947897; doi:10.1371/journal.pone.0196914)
Supplement: S2 Appendix — (DOCX) [file pone.0196914.s002.docx]

**S1 Appendix – Further detail on stakeholder topic prioritisation for the systematic review**

The three potential scenarios for the systematic review are described below with an example research question and the rationale for each.

| **Scenario description** | **Example research question** | **Rationale** |
| --- | --- | --- |
| 1. Evidence for the efficiency and effectiveness of grant peer review in funding systems considered specifically relevant to the UK. | What is the evidence supporting steps to improve the efficiency and effectiveness of research grant peer review relevant to UK research funders? | As a UK researcher funder, NIHR may be particularly interested in research that is either directly relevant, or generalisable, to UK practice. |
| 2. Evidence, from any country, on a variety of peer review innovations that may result in a more efficient and effective peer review system (NB. For this study an innovation was defined as an activity distinct from an existing practice, though it may be assessed in addition to an existing practice). | What empirically examined innovations result in improved efficiency and/or effectiveness of the peer review system in health research funding? | This scenario would bring together all studies, from any country, that have evaluated the effects of implementing particular innovations on the efficiency and effectiveness of the peer review process. Unlike scenario 1, this scenario would focus on outcome evaluation studies that have evaluated the implementation of particular peer review innovations (rather than existing peer review processes) in any country. A wider range of ‘innovations’ would be included in this review than in the review proposed in scenario 1. |
| 3. Evidence, in any country, on innovations to improve the quality of peer review specifically in terms of identification and recruitment of appropriate knowledgeable and skilled reviewers. | What initiatives can potentially improve the quality of peer review, specifically in terms of identification and recruitment of appropriate reviewers? | Identifying and recruiting the knowledgeable and skilled reviewers for each proposal is important. This scenario would capture studies in the map keyworded as investigating ‘Reviewer configurations’, ‘Identification selection and recruitment of peer reviewers’ and ‘Expertise of reviewers’. |

Each scenario was described and discussed in turn and NIHR stakeholders were given the opportunity to ask the research team for more information about the scenario and the evidence from the map pertinent to each. The stakeholders were also given the opportunity of proposing additional scenarios based on the results of the systematic map. The discussion concluded by focusing on three key questions proposed by the research team:

1. Which evidence scenario is the biggest priority currently?
2. How will it help to inform peer review within NIHR?
3. What type of recommendations would be most useful from the systematic review?

Although there was interest from stakeholders in all three scenarios the outcome of the discussion was a clear preference for the second scenario (peer review innovations that may result in a more efficient and effective peer review system). It was considered that a critical evaluation of innovative approaches for improving the efficiency and/or effectiveness of peer review, as covered by this scenario, could help to inform ways of undertaking peer review within NIHR. This consensus was arrived at through discussion rather than a voting process. The stakeholders provided suggestions on issues of particular interest within this scenario (e.g. focusing on evidence only published in the last ten years), enabling the scenario to be revised accordingly. Following the meeting a summary of the discussion was circulated to NIHR working group members not present at the meeting to seek any additional comments. There was no disagreement from any of these other group members on the prioritised scenario.
