# Supplementary material for: Peer review of health research funding proposals: A systematic map and systematic review of innovations for effectiveness and efficiency
Source: PLoS One. 2018 May 11;13(5):e0196914. doi: 10.1371/journal.pone.0196914 (PMC5947897; doi:10.1371/journal.pone.0196914)
Supplement: S1 Protocol — (PDF) [file pone.0196914.s005.pdf]

Wessex Institute (SHTAC & NETSCC), University of Southampton, UK.

Research on Research Project Protocol

A scoping review of studies examining the peer review process of research funding in health

Correspondence to:

Dr Jonathan Shepherd
Principal Research Fellow

Southampton Health Technology Assessments Centre (SHTAC)
University of Southampton
First Floor, Epsilon House, Enterprise Road
The University of Southampton Science Park
Southampton
SO16 7NS
UK

Tel: +44 (0) 23 80 597055
Fax: +44 (0) 23 80 595662

Email: jps@soton.ac.uk

Protocol Version: Final
Date: July 2016

Wessex Institute (SHTAC & NETSCC), University of Southampton, UK.

Executive summary

The peer review of grant applications plays a key role in aiding funding bodies, such as the National Institute for Health Research (NIHR), to allocate funding resources to research proposals that are of the highest scientific merit and that are of the most relevance to policy and practice. The NIHR, through its Push the Pace strategic priority work, which aims to reduce the time needed for each part of NIHR's research pipeline, has identified inconsistencies in the peer review process across the NIHR's funding programmes and a need to determine what a proportionate and fit-for-purpose peer review system would be for the programmes. The NIHR is particularly interested in reducing the time and resources needed. The NIHR's Research on Research (RoR) team is therefore carrying out themed work around peer review to inform the NIHR peer review process. The project proposed here is part of this work, and will be carried out collaboratively by RoR and the Southampton Health Technology Assessments Centre (SHTAC). It will involve a systematic review of research studies that have investigated peer review processes for assessing research funding proposals in health and social care. The review aims to identify methods for ensuring the timely, efficient and good quality peer review of proposals.

A two-stage review is proposed, comprising (1) systematic mapping of the key characteristics of the evidence, followed by (2) a focused synthesis of particular subgroups of studies or topic areas, identified from the map, that NIHR and NIHR Evaluation, Trials and Studies Coordinating Centre (NETSCC) stakeholders deem to be most relevant to NIHR's decision-making needs about the peer review system. A comprehensive search for relevant literature will be undertaken, including searches of academic, electronic bibliographic databases and, in particular, the grey literature (i.e. studies not published in academic journals), where we anticipate that much of the evidence will be located. Search results will be screened on title and abstract for inclusion in the review. We will include studies of any design that investigate any aspect of the peer review of health research grant applications. The characteristics of all the identified, relevant studies will be described in the map, using pre-specified keywords. A report of the mapping exercise findings will be circulated to relevant stakeholders within NETSCC and NIHR for information, and to solicit feedback on areas of the evidence base where a focused synthesis would be most valuable and feasible.

The sets of studies prioritised from the map will then be screened against criteria for inclusion in the focused review. Data will be extracted from the studies meeting the inclusion criteria, using a standard form created for this review. Studies will be critically appraised (to assess their strengths and weaknesses) using a tool suitable to the study's design. One reviewer will carry out inclusion screening and the keywording, and a second reviewer will check the inclusion assessments of and keywords applied to a random sample of studies. One reviewer will extract data from and critically appraise each study, with another reviewer checking the data extractions and critical appraisals. The results of the studies will be tabulated and summarized textually in a narrative synthesis. Where sufficient outcome evaluations of similar interventions and methods are identified these will be considered for quantitative synthesis via meta-analysis. However, it is anticipated that there will be few such studies. From the review, we will make recommendations regarding processes and methods, which have been found to promote timely, efficient, and good quality peer review, which could be implemented within the NIHR. Recommendations for further methodological research will also be made, where possible, to inform the RoR programme.

Wessex Institute (SHTAC & NETSCC), University of Southampton, UK.

1. Background and rationale

Peer review is a key aspect of quality assurance in academic research. It is used to reassure research funders, such as the National Institute for Health Research (NIHR), that research proposals are of the highest scientific merit and that potentially funded research is appropriate to policy and practice needs. Peer review can also be employed at later stages of the research lifecycle, such as ensuring the scientific credibility of research outputs. There is a need to ensure an effective and efficient infrastructure for peer review to support high quality research in health.

Push the Pace is one of the NIHR strategic priorities and was originally established in April 2014. The objective of Push the Pace was “A reduction in time of the NIHR components of the research pipeline, including time metrics, aiming for a reduction of 20% in the NIHR lifecycle.” In November 2015, a Push the Pace report was presented at the NIHR Strategy Board, which made a number of recommendations for endorsement by the NIHR Strategy Board. One of these recommendations was “Determine what a proportionate peer review system for applications is, which is fit for purpose to meet NIHR’s needs.”

Work conducted under Push the Pace, found that peer review does not appear to be proportionate across the NIHR research programmes. The inconsistencies found between the different NIHR programmes appear to have no apparent cause or benefit. With peer review being a key part of the research lifecycle, it is estimated that one to two months may be saved if a more streamlined approach was to be implemented by NIHR programmes.

It is on this basis that the NETSCC Research on Research (RoR) programme was asked to assess peer review at the funding stage of the research lifecycle. There are a number of issues around the peer review of research funding process that are of interest to NIHR, including the optimum number of reviewers to approach, the type of experts from whom reviews are sought and the nature of the task they are asked to do, methods to solicit and collate reviewers’ comments, the role of incentives for peer reviewers, involvement of patients and public, timing, and quality. At present there is particular interest in reducing the time and resources needed for peer review, whilst maintaining and improving quality standards.

As part of the RoR themed activity around peer review, a systematic review will be conducted and the results will be reported back to the NIHR Push the Pace strategic priority. The research contributes to the ‘Adding Value in Research’ framework in terms of Pillar 1 ‘Improve the design and planning at the application stage.’

1.1 Current evidence base

Our initial literature searches have identified 1784 unique references published from 1970 to 2016. The volume of literature has increased through time, with the majority of these references (88%) having been published since the start of 1990 (Figure 1).

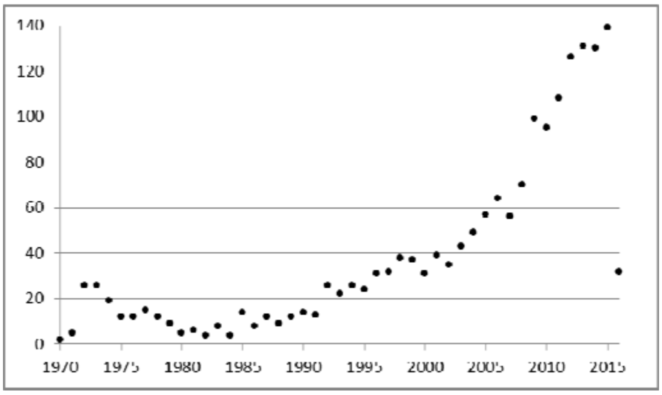

Figure 1 Distribution of publication dates of references identified in scoping searches

An initial pilot test of the draft eligibility criteria (see section 6.2 below) on a subset of the references published during 2015 (n=134) and 2016 (n=32) suggests that approximately 12% of the identified literature (i.e. 20 of these 166 references[1-20]) would be eligible for inclusion in the systematic map.

The studies identified in the pilot eligibility screening have addressed various different aspects of the peer review of research grant applications. These include (among others): investigating impacts of simplifying the peer review process;[1,7] investigating impacts of different approaches for assigning reviewers; [11] determining the optimal number of reviewers;[16] investigating agreement between individuals or groups of reviewers,[14] or between different review panels;[3] assessing the importance of review panel discussions;[2,19] and exploring types of bias that occur during peer review.[13,17,18] Studies have also investigated approaches for training reviewers to improve consistency,[15] and proposed methods to assist the selection of research projects.[20] Several studies have explored whether the scores or rankings resulting from peer review are predictive of grant proposal funding success[8,9] or of the subsequent impact of funded projects.[4,5,12] Other aspects of peer review that have been assessed are monetary costs[6] and how the peer review process influences innovation.[17]

The majority of studies appear to have conducted analyses of retrospective data.[2,4,5,8,9,12,14,18,19] Some studies have employed prospective experimental approaches,[3,7,14] simulation and/or modelling approaches, [11,16,17] or were based on observational evidence.[1]

We have also identified an existing Cochrane systematic review, published in 2007,[21] which aimed to assess the impact of different peer review processes for assessing grant applications on the quality of the funded research. The review only included comparative studies (i.e. those which compared two or more peer review methods). The authors of the review searched a range of academic bibliographic databases up to June 2002 and supplemented this with searches of relevant books and journals, as well as the reference lists of included studies and relevant reviews. They identified 10 studies for inclusion in the review. None provided information about the impact of different peer review processes on the quality of the research funded. Instead the authors synthesised the outcomes measured in the studies (agreement between reviewers, approval rates and priority scores allocated to the proposals). The review found that when simplified peer review processes were compared with standard processes, there was little impact on the outcome of peer review. A number of the studies examined the agreement of reviewers' decisions when using different reviewers, review panels or types of reviewers. These studies resulted in mixed findings

about the level of reviewer agreement, likely due to the wide range of settings and peer review situations across the studies. One study that compared blinded and open peer review suggested that open peer review results in more bias. Overall, the reviewers concluded that the quality of the evidence base was questionable and that there was a strong need for experimental studies that examine the impact of different peer review processes on the quality of funded research.

This Cochrane review is now out-of-date, as the searches were conducted up to 2002, pointing to the need for a new, up-to-date review of the evidence base. Our proposed review will address this need. In our review, we will build on the Cochrane review by searching the grey literature – which was not searched for the Cochrane review. We anticipate that much of the relevant evidence will be in the grey literature (e.g. reports available on the internet sites of organisations that commission or produce health research). The proposed review will additionally be wider in scope than the Cochrane review, as it will include a range of study types, including experimental, observational and non-interventional studies (e.g. surveys and interview studies), rather than focusing solely on comparative intervention studies. It is likely that these other types of research (such as surveys of researchers' views of the peer review system) will provide insights into the peer review process that may have implications for NIHR procedures, that complement the insights gained from comparative studies. Including these studies may also widen the pool of studies available for synthesis in the review. The proposed review will additionally be wider in scope than the Cochrane review, as it will initially focus on any aspect of the peer review process and any measured outcomes, through the incorporation of systematic mapping to characterise the overall evidence base (see Section 5.2). We will share the map with NIHR and NETSCC stakeholders to gain their feedback on which sets of studies or topic areas identified in the map could be the focus of the in-depth synthesis, so that the review can best meet the needs of the NIHR.

2. Research question

What is the research evidence on methods and processes for timely, efficient and good quality peer review of research funding proposals in health?

3. Implications for NIHR / NETSCC / NETS programmes

Please refer to above section 2.

4. Aims and Objectives

Aim
To identify methods to ensure timely, efficient, good quality peer review of research funding proposals in health to inform peer review within the NIHR.

Objectives
1. To develop a systematic map to identify and characterise methodological studies and reports on the peer review of research funding proposals in health.
2. To undertake focused synthesis of subgroups of studies deemed to be of most relevance to the needs of key stakeholders in NIHR and NETSCC, including critical assessment of the strengths and limitations of the evidence and, potentially, exploration of facilitators and barriers or other process indicators.

3. To make conclusions and recommendations on effective methods for peer review of research funding proposals in health that could potentially be implemented within NIHR.
4. To make conclusions and recommendations for further methodological research on methods for the peer review of research funding proposals in health.

5. Methods

A two-stage evidence synthesis is proposed, comprising (1) systematic mapping of the key characteristics of the evidence, followed by (2) a focused synthesis of particular areas to answer specific research questions (Figure 2). This is a flexible and pragmatic approach to evidence synthesis that has been successfully applied in a number of published systematic reviews of complex health and education interventions as a means of characterising the evidence base to facilitate a focused policy-relevant synthesis. [22-26]

5.1 Literature Searching

A comprehensive search for relevant literature will be undertaken by an experienced information specialist in health. Search sources will include key health bibliographic databases, relevant internet sites, contact with experts in the field, and checking of reference lists of relevant articles.

Electronic bibliographic databases (and platform) to be searched:

- Medline (Ovid)
- Medline In-Process (Ovid)
- Embase (Ovid)
- The Cochrane Library (Cochrane Database of Systematic Reviews; Cochrane Central Register of Controlled Trials (CENTRAL); Cochrane Methodology Register)
- Psychinfo (Ebsco)
- Social Science Citation Index (Web of Science)
- Delphis (University of Southampton Library database)

It is anticipated that much of the relevant evidence for this topic will be located in the grey literature (i.e. not formally published in academic journals). Particular emphasis will therefore be given to searching internet sites of organisations that commission or produce health research to identify studies they may have conducted on peer review. These are:

Wessex Institute (SHTAC & NETSCC), University of Southampton, UK.

Figure 2 - Key stages of the project

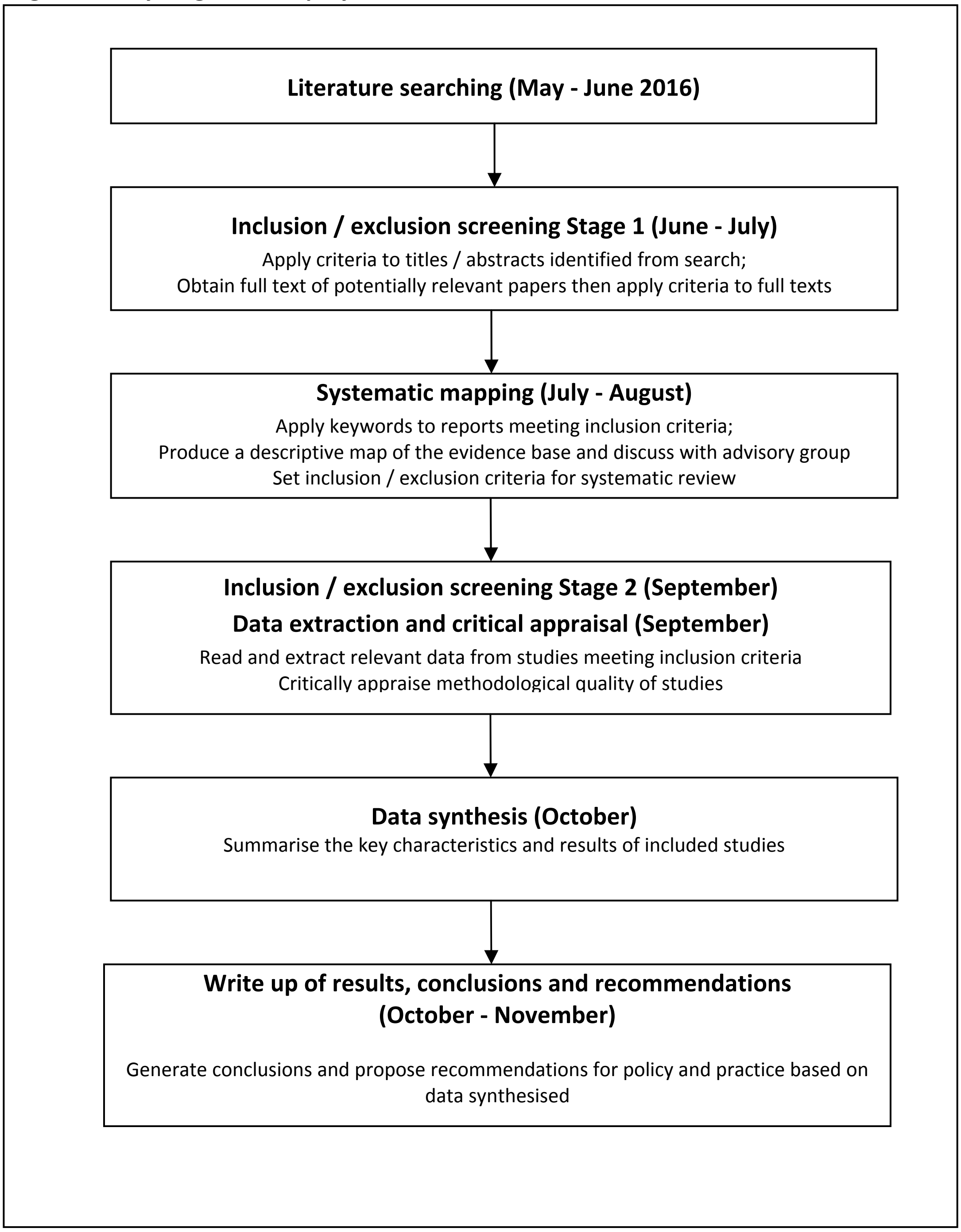

Arthritis Research UK
Association of Medical Research Charities
Biotechnology and Biological Sciences Research Council (BBSRC)
British Academy
British Heart Foundation
Campbell Collaboration
Cancer Research UK

Wessex Institute (SHTAC & NETSCC), University of Southampton, UK.

Centers for Disease Control (CDC) USA
Center for Health Services Research (CHSR) USA
Chief Scientist Office Scotland
Cochrane Methodology Review Group
Department of Health England
Department for International Development (DFID)
Diabetes UK
EPPI Centre
European Research Council
Economic and Social Research Council (ESRC)
Gates Foundation
Google Scholar
Global Research Council
Health and Care Research Wales
HSC Public Health Agency (NI)
3iE (International Initiative for Impact Evaluation)
Joseph Rowntree Foundation
Kings Fund
Medical Research Council (MRC)
National Academies of Sciences (Health and Medicine division)
National Health & Medical Research Council Australia (NHMRC)
National Institute for Health (NIH) USA
National Institute for Health Research (NIHR)
National Science Foundation
Nuffield Foundation
Parliamentary Office of Science and Technology (POST)
Public Health England
Research Councils UK
RIN Research Information Network
Royal Society
Royal Society of Medicine
The Health Foundation
The Leverhulme Trust
The RAND Corporation
The Wellcome Trust
World Health Organisation (WHO)

The searches will identify international literature, but will be limited to references in the English language.

5.2 Inclusion criteria for Stage 1 - systematic map

The initial inclusion criteria will be broad, to permit classification of a wide range of evidence of potential relevance to the NIHR. To be included references need to report:

- A research study, of any design. Given the broad initial scope of this project study designs will vary according to the type of research question being posed, and could include experimental designs (e.g. random or non-randomised controlled trials), observational studies (e.g. cohort studies), or non-interventional designs (e.g. surveys, interviews, focus groups, consensus-setting exercises). Systematic reviews of the above studies will also be included.
- An investigation into any aspect of the peer review of health research funding.

Health is defined in a broad sense, to include research that is relevant to the scope of NIHR, including health and social care, and public health and health promotion.

References reporting investigations into the peer review of research outputs will not be included (unless they also report peer review of funding proposals) as this is a process that is distinct from peer review of funding proposals, and would be more appropriate as a separate project.

Although the draft eligibility criteria propose that editorials and commentaries are not eligible (since in general they do not directly report research evidence), some of these references might provide contextual information that might help in identifying relevant evidence. Therefore, although editorials and commentaries will not be included in the systematic map, we will check any that are identified in case they link to relevant research evidence and attempt to identify publications relating to the research evidence mentioned.

Each title and abstract will be screened by independently by two reviewers. If agreement between reviewers cannot be reached a third reviewer will be consulted. Full texts of references deemed relevant will be retrieved for screening. All full texts will be screened by one reviewer and checked by a second. Again, a third reviewer will be consulted in cases of disagreement.

5.3 Production of the systematic map

All studies meeting the inclusion criteria described above in Section 5.2 will be classified through the systematic application of pre-specified keywords. A draft list of descriptive keywords will be developed based on a pilot analysis on a subset of the studies, to capture information about relevant study characteristics (e.g. their scope and methodology). A logic model will be devised as part of this process to conceptualise the mechanisms and resources needed for peer review of funding applications, the activities and the intended outputs and outcomes.[27, 28] A visual schematic will be used to illustrate the relationships between the components of the logic model (e.g. inputs, activities, outputs and outcomes).

The keyword list will be updated in light of feedback from the project team and stakeholders in NETSCC and NIHR then applied systematically to code all references meeting the inclusion criteria. The keywords would cover relevant aspects of:

- the scope of the studies (e.g. identification and selection of peer reviewers; incentives for peer reviewers; costs and time; timing of peer review; quality assurance and

accuracy of peer review; solicitation and collation of reviewer feedback; ethics; handling competing interests)
- study population (e.g. researchers, peer reviewers (academic and lay), grant awarding organisations, public)
- study design (e.g. experimental, observational, non-intervention)
- study context (e.g. country; type of research funder; topic area of research funding)
- study measures, including outcomes (from outcome evaluations), process measures (from process evaluations), issues investigated (from surveys, interview studies etc) (NB. The keywording will not, however, characterise the results of studies. Results will be synthesised in stage 2 – see below Section 5.4).

Each study will be keyworded by one reviewer and a random sample checked by a second. The resulting reference coding, collated systematically in a relational database (e.g. in Microsoft Excel), will be used to produce a descriptive map of the characteristics of the evidence. The map will be presented in a report using tables, figures and text.

The map report will be circulated to relevant stakeholders within NETSCC and NIHR for information, and to solicit feedback on areas of the evidence base (in terms of subsets of studies identified by their keywords) where a focused synthesis would be of most value and would be feasible (i.e. where sufficient evidence exists). Feedback will be sought electronically, and/or face-to-face in a meeting or specially convened workshop. Progress updates will be reported at Push the Pace meetings as part of the wider peer review themed activity conducted under the RoR programme.

Where evidence allows we will present stakeholders with options for different sets of studies with selected characteristics, which the synthesis could potentially focus on. The sets of studies will be presented as contrasting 'scenarios'. Stakeholders may wish to select sets of studies according to the following characteristics:
- Focus relevance: such as the efficiency of peer review (reducing the amount of time and costs)
- Study design: where the outcomes of peer review processes have been evaluated research designs accepted to have greater rigour might be prioritised for inclusion (e.g. experimental designs)
- Context: studies conducted of funding programmes that most closely resemble the NIHR's research portfolio could be prioritised for inclusion (e.g. research commissioned to inform health policy, practice and future research).

5.4 Inclusion criteria for Stage 2 - synthesis

A set of studies will be assembled from the systematic map, identified according to the keywords that represent the area of focus agreed on with stakeholders in Stage 1, above. A set of inclusion criteria will be devised, based on the area of focus. The studies from the map will be screened against these criteria in detail. The purpose of this exercise is to ensure, through closer examination than was possible in stage 1, whether the identified studies adequately address the agreed area of focus. The application of inclusion criteria is therefore a formal process to ensure a transparent and systematic synthesis. Each full text will be screened by one reviewer and checked by a second.

5.5 Data extraction of studies included in the synthesis

Each study meeting the inclusion criteria for the synthesis will be read by one researcher and will have relevant data extracted from it into a template designed specifically for this study. A second researcher will check the data for accuracy and interpretation, and any disagreements resolved through discussion.

5.6 Critical appraisal of studies included in synthesis

Due to the diverse range of potentially eligible studies a number of different critical appraisal instruments will be needed, depending on the types of studies to be included in the synthesis. Experimental studies, such as RCTs or non-randomised controlled trials, will be appraised using Cochrane Risk of Bias criteria (including modification of these criteria for non-randomised studies), a widely used instrument in Cochrane and non-Cochrane systematic reviews.[29] The quality of process evaluations and qualitative studies will be assessed by a specially devised instrument by Harden and Gough (2012).[30] These criteria assess whether steps were taken to minimise bias and error in sampling, data collection procedures, and data analysis. They also assess whether the findings are grounded in the data, and overall whether the findings presented are reliable and useful

Each study will be critically appraised by one researcher and checked by a second.

5.7 Synthesis methods

The results of the studies will be tabulated and summarized textually in a narrative synthesis. Where sufficient outcome evaluations of similar interventions and methods are identified these will be considered for quantitative synthesis via meta-analysis. However, it is anticipated that there will be few such studies.

The synthesis will take into consideration the generalisability of the evidence from international studies to the UK, in terms relevance to the NIHR. Where possible, stakeholder input will be sought to ensure that the conclusions and recommendations made are relevant and meaningful to NIHR research programmes as possible.

Recommendations will be made regarding any processes and methods which have been found to promote timely, efficient, good quality peer review, in accordance with the aims of this project. Recommendations for further methodological research will also be made, where possible, to inform the RoR programme.

6. Declaration of interests

The members of the project team declare no competing interests.

7. Access to data

All electronic data from the project will be stored in a University of Southampton network drive within the SHTAC group folder (J drive). This is only accessible to SHTAC staff, and any additional staff who are granted access to the project folder.

Wessex Institute (SHTAC & NETSCC), University of Southampton, UK.

8. Protocol amendments

Any amendments required to the protocol will be discussed within the project team and where necessary an amended protocol will be produced, with a clear statement of the changes made and the rationale for them. The final report will include a section reporting any changes made to the protocol.

9. Dissemination

The key outputs of the project will include:

- A final report to NETSCC/NIHR. This will be a full account of the scope, methods and results of the project.
- One or more articles submitted to a relevant peer review journal reporting the findings from the review (e.g. Journal of Health Services Research & Policy; Journal of Epidemiology and Community Health) and also the methodology used (e.g. BMC Medical Research Methodology).
- Presentation internally to NETSCC/NIHR, and externally within the University (e.g. Faculty of Medicine seminar), or at relevant national and international conferences (e.g. Evidence Live; Cochrane Colloquium, 2017 International Peer Review Congress).

10. Project Management

10.1 Project Team

Table 1: Project team members

**Team member**	**Role**	**% of time available**	**Project tasks team member will be involved in**
Professor Jeremy Wyatt, Wessex Institute	Principal Investigator	10	Strategic oversight of the scope and methods of the review
Dr Jonathan Shepherd, SHTAC	Researcher	10	Overall project co-ordination, methodological input (e.g. design of criteria, instruments, piloting etc). Inclusion/exclusion screening, keyword application, synthesis, write-up of findings
Dr Geoff Frampton, SHTAC	Researcher	30	Inclusion/exclusion screening, keyword application, synthesis, write-up of findings
Dr Karen Pickett, SHTAC	Researcher	30	Inclusion/exclusion screening, keyword application, synthesis, write-up of findings
Amanda Blatch-Jones, NETSCC	Researcher	10	RoR management, advice and support via NIHR PtP2 workstream, progress feedback into PtP2/NETS/RoR business for further recommendations

Wessex Institute (SHTAC & NETSCC), University of Southampton, UK.

Team member	Role	% of time available	Project tasks team member will be involved in
			Inclusion/exclusion screening, keyword application, synthesis, write-up of findings

10.2 Project timetable

Milestone	Date
Stage 1	
Literature searches	May - June
Inclusion screening for descriptive map	June - July
Application of keywords	July - August
Completion and circulation of descriptive map	July - August
Consultation on map results and focus for stage 2	September
Stage 2	
Inclusion / exclusion screening	September
Data extraction and critical appraisal	September
Synthesis	October
Discussion of preliminary conclusions and recommendations	October - November
Final report	November

11. References

1. Barnett AG, Herbert DL, Campbell M, et al. Streamlined research funding using short proposals and accelerated peer review: an observational study. BMC Health Serv Res 2015;**15**:55.
2. Carpenter AS, Sullivan JH, Deshmukh A, et al. A retrospective analysis of the effect of discussion in teleconference and face-to-face scientific peer-review panels. BMJ Open 2015;**5**(9):e009138.
3. Clarke P, Herbert D, Graves N, et al. A randomized trial of fellowships for early career researchers finds a high reliability in funding decisions. J Clin Epidemiol 2016;**69**:147-51.
4. Doyle JM, Quinn K, Bodenstein YA, et al. Association of percentile ranking with citation impact and productivity in a large cohort of de novo NIMH-funded R01 grants. Molecular Psychiatry 2015;**20**(9):1030-36.
5. Fang FC, Bowen A, Casadevall A. NIH peer review percentile scores are poorly predictive of grant productivity. elife 2016;**5**.
6. Geuna A, Piolatto M. Research assessment in the UK and Italy: Costly and difficult, but probably worth it (at least for a while). Research Policy 2016;**45**:260-71.
7. Herbert DL, Graves N, Clarke P, et al. Using simplified peer review processes to fund research: a prospective study. BMJ Open 2015;**5**(7):e008380.

8. Hume KM, Giladi AM, Chung KC. Factors impacting successfully competing for research funding: an analysis of applications submitted to the Plastic Surgery Foundation. Plast Reconstr Surg 2015;**135**(2):429e-35e.
9. Kaatz A, Magua W, Zimmerman DR, et al. A quantitative linguistic analysis of National Institutes of Health R01 application critiques from investigators at one institution. Acad Med 2015;**90**(1):69-75.
10. Li D, Agha L. Research funding. Big names or big ideas: do peer-review panels select the best science proposals? Science 2015;**348**(6233):434-8.
11. Li L, Wang Y, Liu G, et al. Context-Aware Reviewer Assignment for Trust Enhanced Peer Review. PLoS ONE 2015;**10**(6):e0130493.
12. Lindner MD, Nakamura RK. Examining the Predictive Validity of NIH Peer Review Scores.[Erratum appears in PLoS One. 2015;10(6):e0132202; PMID: 26121031]. PLoS ONE 2015;**10**(6):e0126938.
13. Mutz R, Bornmann L, Daniel H-D. Testing for the fairness and predictive validity of research funding decisions: A multilevel multiple imputation for missing data approach using ex-ante and ex-post peer evaluation data from the Austrian science fund. Journal of the Association for Information Science & Technology 2015;**66**(11):2321-39.
14. Pina DG, Hren D, Marusic A. Peer Review Evaluation Process of Marie Curie Actions under EU's Seventh Framework Programme for Research. PLoS ONE 2015;**10**(6):e0130753.
15. Sattler DN, McKnight PE, Naney L, et al. Grant Peer Review: Improving Inter-Rater Reliability with Training. PLoS ONE 2015;**10**(6):e0130450.
16. Snell RR. Menage a quoi? Optimal number of peer reviewers. PLoS ONE 2015;**10**(4):e0120838.
17. Sobkowicz P. Innovation suppression and clique evolution in peer-review-based, competitive research funding systems: An agent-based model. JASSS 2015;**18**(2):1-23.
18. van der Lee R, Ellemers N. Gender contributes to personal research funding success in The Netherlands. Proc Natl Acad Sci U S A 2015;**112**(40):12349-53.
19. Vo NM, Trocki R. Virtual and Peer Reviews of Grant Applications at the Agency for Healthcare Research and Quality. South Med J 2015;**108**(10):622-6.
20. Zhu WD, Liu F, Chen YW, et al. Research project evaluation and selection: an evidential reasoning rule-based method for aggregating peer review information with reliabilities. Scientometrics 2015;**105**(3):1469-90.
21. Demicheli V, Di Pietrantonj C. Peer review for improving the quality of grant applications. The Cochrane database of systematic reviews 2007(2):Mr000003.
22. Frampton GK, Harris P, Cooper K, et al. Educational interventions for preventing vascular catheter bloodstream infections in critical care: evidence map, systematic review and economic evaluation. Health technology assessment (Winchester, England) 2014;**18**(15):1-365.
23. Miake-Lye IM, Hempel S, Shanman R, et al. What is an evidence map? A systematic review of published evidence maps and their definitions, methods, and products. Systematic Reviews 2016;**5**(1):1-21.
24. Schucan Bird K, Newman M, Hargreaves K, et al. Workplace-based learning for undergraduate and pre-registration healthcare professionals: A systematic map of the UK research literature 2003-2013. London: EPPI-Centre, Social Science Research Unit, UCL Institute of Education, University College London., 2015.
25. Shepherd J, Kavanagh J, Picot J, et al. The effectiveness and cost-effectiveness of behavioural interventions for the prevention of sexually transmitted infections in

young people aged 13-19: a systematic review and economic evaluation. Health technology assessment (Winchester, England) 2010;**14**(7):1-206, iii-iv.
26. Wang DD, Shams-White M, Bright OJ, et al. Creating a literature database of low-calorie sweeteners and health studies: evidence mapping. BMC medical research methodology 2016;**16**:1.
27. Anderson LM, Petticrew M, Rehfuess E, et al. Using logic models to capture complexity in systematic reviews. Research synthesis methods 2011;**2**(1):33-42.
28. W.K. Kellogg Foundation. Logic Model Development Guide: Using Logic Models to Bring Together Planning, Evaluation and Action (Updated January 2004). Battle Creek, 2004.
29. Higgins JPT, Altman DG, Gøtzsche PC, et al. The Cochrane Collaboration's tool for assessing risk of bias in randomised trials. BMJ 2011;**343**.
30. Harden A, Gough D. Quality and relevance appraisal. In: Gough D, Oliver S, Thomas J, eds. An introduction to systematic reviews. London: Sage, 2012:152-78.

12. Appendices

12.1 Medline search strategy

1 "peer review"/ or peer review, research/ (12076)
2 (peer adj review*).tw. (18395)
3 1 or 2 (26600)
4 (grant* adj2 (financ* or budget* or allocat*)).tw. (151)
5 (research adj2 (fund* or grant* or proposal* or application* or applicant* or submission* or budget* or financ*)).tw. (14327)
6 (program* adj grant*).tw. (128)
7 (grant adj2 (application* or applicant* or submission*)).tw. (511)
8 (grant adj2 proposal*).tw. (257)
9 (grant adj2 award*).tw. (201)
10 Financing, Organized/ or Financing, Government/ (25154)
11 Research Support as Topic/ec (2539)
12 (fund* and decision*).tw. (9348)
13 (grant* and decision*).tw. (1241)
14 (protocol* adj5 (grant* or fund*)).tw. (352)
15 Research Support as Topic/ec (2539)
16 Financial Management/ (15951)
17 or/4-16 (65714)
18 3 and 17 (1106)
19 ("peer review*" and grant*).ti. (65)
20 ("peer review*" and fund*).ti. (35)
21 18 or 19 or 20 (1120)
22 ("peer review" and process* and research and grant*).tw. (67)
23 ("peer review" and process* and research and fund*).tw. (125)
24 21 or 22 or 23 (1173)
25 remove duplicates from 24 (1160)
26 from 25 keep 1-1000 (1000)
27 from 25 keep 1001-1160 (160)
