# Supplementary material for: Peer review of health research funding proposals: A systematic map and systematic review of innovations for effectiveness and efficiency
Source: PLoS One. 2018 May 11;13(5):e0196914. doi: 10.1371/journal.pone.0196914 (PMC5947897; doi:10.1371/journal.pone.0196914)
Supplement: S1 Table — (DOCX) [file pone.0196914.s006.docx]

**EFFICIENCY OUTCOMES**

**S1A Table Reviewer agreement reported in 2-group studies**

| **Study** | **Outcome** | **Innovation** | **Comparator** | **Effect** |
| --- | --- | --- | --- | --- |
| Gallo et al. 2013; Carpenter et al. 2015 | Intraclass correlation coefficient (ICC) and inter-rater reliability (IRR) | ***Teleconference*** (2 years:2011-2012) | ***Face-to-face meeting*** (2 years: 2009-2010) | ICC ranged 0.84 to 0.87 over the 4 study years (stated SE was approximately 0.06 over all years; p<0.01). IRR was 0.98 for all years. Authors concluded peer review had high reliability; they stated variation in ICC across years was less than the calculated error and there was no obvious time trend. In both groups reliability between reviewers increased post-discussion. |
| Sattler et al. 2015 (RCT) | Proportion with correct selection of rating scale values | ***Reviewer training video***  74%  Novice reviewers had slightly higher accuracy (79%) than experienced reviewers (69%) (presented in chart only; data extracted using Enguage digitizer) | ***No-training group***  35%  Novice reviewers had slightly lower accuracy (33%) than experienced reviewers (40%) (presented in chart only; data extracted using Enguage digitizer) | Not reported. Appears to be the mean of novice and experienced reviewers, which are indicated separately in a chart in the paper. Stated that reviewer experience had no direct influence and did not interact with the group (no data reported). |
|  | Inter-rater reliability estimated by intra-class correlation coefficient (r_icc_) | ***Reviewer training video***  0.89 (95% CI 0.71 to 0.99) | ***No-training group***  0.61 (95% CI 0.32 to 0.96) | Difference was significant (linear regression p<0.05). |

**S1B Table Reviewer agreement reported in single-group studies**

| **Study** | **Outcome** | **Innovation aspects assessed** | **Effect** |
| --- | --- | --- | --- |
| Fleurence et al. 2014 | Agreement in scores between and within reviewer groups (scientists, patients, stakeholders), and before and after discussion | Merit-review score agreement for lead reviewers (n=4) before discussion | Score scatter plots (scientists vs patients or scientists vs stakeholders) indicated ‘no meaningful relation’ between the scores of each group for both comparisons (no statistics reported) |
|  |  | Merit-review score agreement for lead reviewers (n=4) after discussion | Score scatter plots (scientists vs patients or scientists vs stakeholders) indicated ‘mild association’ among scores of each group for both comparisons (no statistics reported) |
|  |  | Merit-review score agreement for all reviewers (n=21), before discussion | Not reported |
|  |  | Merit-review score agreement for all reviewers (n=21) after discussion | Score scatter plots (scientists vs patients or scientists vs stakeholders) and also Bland-Altman plots indicated a ‘slight positive linear trend’ for both comparisons (limits of agreement were relatively narrow: -14 to 14 for scientists vs patients, and -11 to 10 for scientists vs stakeholders) |
|  |  | Merit-review score agreement before and after discussion within each reviewer group | Score scatter plots (pre-discussion vs post-discussion) showed ‘consistent positive linear relationships’ for all three reviewer groups. However, variability in pre-post score differences, indicated by 95% limits of agreement in Bland-Altman plots, was higher for patient reviewers (-27 to 20) than for scientists (-21 to 15) or stakeholders (-22 to 16). Basic percentage change data suggested that patients more frequently changed their score decision, either positively or negatively, than scientists or stakeholders, by ≥10 points, but no statistics are reported. |
| Holliday et al. 2010 | Reviewer agreement | 3-round modified Delphi process (n=5 reviewers) | Authors state that no reviewers recorded any objections at the end of Round 1 or Round 2 but do not mention reviewer agreement after Round 3, although the abstract states that the four highest-ranking applications were recommended for funding ‘with agreement from reviewers’. They also state that the widest range of scores (i.e. least agreement) was observed in Round 1 (which assessed scientific merit). No statistics are reported (mean scores per applicant are reported but without variance estimates). The authors don’t say what ‘objections’ refers to – it appears to mean any part of the process, including any disagreements between reviewers, although this is not explicit. Also, one reviewer said discussion would have been beneficial (Table 2 in paper), ‘especially on grants where there was a clear difference in opinion’. This suggests there was some lack of reviewer agreement, but unclear at what part of the process. |

**S1C Table Peer review time reported in 2-group studies**

| **Study** | **Outcome** | **Innovation** | **Comparator** | **Effect** |
| --- | --- | --- | --- | --- |
| Gallo et al. 2013; Carpenter et al. 2015 | Average peer review discussion time (mins per application; not stated but presumed to be mean ± SD) | ***Teleconference***  2011: 19 ± 0.8 (19 panels)  2012: 22 ± 1.3 (13 panels)  Overall 2011-12: 20 ± 0.7  ***Pilot video teleconference (methods not reported)*** 2011 (2 panels, with 6 or 9 applications): average time 15 and 17 minutes | ***Face-to-face meeting***  2009: 23 ± 0.9 (20 panels)  2010: 29 ± 1.4 (13 panels)  Overall 2009-10: 23.9 ± 0.7 | *Overall difference, face-to face vs teleconference:* F_3,61_=14.54; P<0.001  *Specific mean differences, face-to-face vs teleconference:*  *2010 vs 2011:* 9.97 (95% CI 5.56 to 14.37; P<0.001)  *2010 vs 2012:* 7.43 (95% CI 2.63 to 12.24; P<0.001) |
| Herbert et al. 2015 | Peer review time per proposal, hours (for n proposals) | ***7-person simplified panel***  Basic science: 4.3 (n=36)  Public health: 3.5 (n=36)  Overall mean: 3.9 (n=72)  ***2-person ‘journal’ panel***  Basic science: 4.7 (n=36)  Public health: 2.4 (n=36)  Overall mean: 3.6 (n=72) | ***12-person standard panel***  Not reported other than an average of 91 proposals was assessed in a 1-week-long meeting (43 panels with 12 members) (authors were contacted and confirmed that no further data on the comparator are available) | Not reported |
|  | Time spent on spokesperson report, per proposal, hours (for n proposals) | ***7-person simplified panel***  Basic science: 2.2 (n=36)  Public health: 1.2 (n=36)  Overall mean: 1.7 (n=72) | ***12-person standard panel***  Not reported | Not reported |
| Sattler et al. 2015 | Mean time taken to read grant review criteria ± SD, mins | ***Reviewer training video***  6.1 ± 4.8 | ***No-training group***  4.2 ± 4.8 | Poisson regression z=2.17; P=0.03 |
| Vo et al. 2015 | Total meeting length, mins, and length per special emphasis panel (SEP) or study section (SS) (for n proposals) | ***WebEx videoconference***  2580 (n=128) (6 sessions)  SS1: 450 (n=22)  SS2: 540 (n=34)  SS3: 210 (n=16)  SS4: 495 (n=16)  SEP1: 225 (n=6)  SEP2: 660 (n=34) | ***Face-to-face meeting***  2937 (n=113) (5 sessions)  (data not reported separately per SS or SEP session) | Not reported (difference = 357 min) |
|  | Average discussion time per proposal, mins, and per special emphasis panel (SEP) or study section (SS) (for n proposals) | ***WebEx videoconference***  20 (n=128) (6 sessions)  SS1: 22 (n=22)  SS2: 16 (n=34)  SS3: 13 (n=16)  SS4: 31 (n=16)  SEP1: 37 (n=6)  SEP2: 19 (n=34) | ***Face-to-face meeting***  26 (n=113) (5 sessions)  (data not reported separately per SS or SEP session) | Not reported (difference = 6 min)  (note: data are as reported in the publication but panel and overall averages in the videoconference group do not quite agree; based on the individual session data the overall mean would be 23 or the median 20.5) |

**S1D Table Peer review time reported in single-group studies**

| **Study** | **Outcome** | **Innovation aspects assessed** | **Effect** |
| --- | --- | --- | --- |
| Barnett et al. 2015 | Average peer review time per proposal, mins (4 funding rounds with 31 to 74 eligible proposals) | ***Streamlined (short) proposal with accelerated peer review***  Initial review: 36 (range 15 to 105)  Face-to-face meeting: 10 (range not reported) | No effects were tested. Not reported whether data are mean or median. |
|  | Average time from proposal submission to funding outcome notification, weeks (for n eligible proposals) | ***Streamlined (short) proposal with accelerated peer review***  Funding round 1 (2012): 6 (n=74)  Funding round 2 (2012): 8 (n=89)  Funding round 3 (2013): 7 (n=34)  Funding round 4 (2013): 8 (n=31) | No effects were tested. Not reported whether data are mean or median. Paper states that successful research teams were notified within 2 weeks of interview, which was a maximum of 8 weeks after proposal submission. |

**S1E Table Costs of peer review reported in 2-group studies**

| **Study** | **Outcome** | **Innovation** | **Comparator** | **Effect** |
| --- | --- | --- | --- | --- |
| Herbert et al. 2015 | Cost of peer review per proposal, Australian dollars (for n proposals): (a) reviewer salary cost; (b) spokesperson report salary cost; (c) attendance expenses; (d) total cost | ***7-person simplified panel***  Basic science proposals (n=36):  (a) 434; (b) 204; (c) 548; (d) 1186  Public health proposals (n=36):  (a) 390; (b) 115; (c) 525; (d) 1030  Mean for all proposals (n=72):  (a) 412; (b) 160; (c) 537; (d) 1109  ***2-person ‘journal’ panel***  Basic science proposals (n=36):  (a) 465  Public health proposals (n=36):  (a) 252  Mean for all proposals (n=72):  (a) 359 | ***12-person standard panel***  Not measured directly, but the authors estimated total peer review costs in 2009 to be 4.44 million Australian dollars for 2983 proposals (based on a previous study; reference provided); when inflated to 2013 prices they estimated the standard panel process would cost 6.3 million Australian dollars for 3821 proposals. The estimated cost of reviewing the same number of proposals was 4.2 million Australian dollars using 7-person simplified panels and 1.4 million Australian dollars using 2-person ‘journal’ panels. | Not measured directly, but based on the estimated costs of peer review the cost savings per year compared to the 12-person standard panel would be 2.1 million Australian dollars for 7-person simplified panels and 4.9 million Australian dollars for 2-person ‘journal’ panels. |
| Vo et al. 2015 | Cost of peer review per reviewer per day, US dollars | ***WebEx videoconference***  (6 sessions, 128 proposals)  324 | ***Face-to-face meeting***  (5 sessions, 113 proposals)  1314 | Cost saving of approximately 1000 US dollars per reviewer per day |

**S1F Table Optimal number of reviewers reported in a 2-group study**

| **Study** | **Outcome** | **Innovation** | **Comparator** | **Effect** |
| --- | --- | --- | --- | --- |
| Mayo et al. 2006 | Number of reviewers required to achieve consistent proposal ranking: (a) for minimum acceptable consistency (Cronbach’s alpha ≥0.7); (b) for optimal consistency (Cronbach’s alpha ≥0.8) | ***‘Ranking’ method***  New investigator proposals:  (a) 10; (b) not reached  New team proposals:  (a) 5; (b) 8  Overall (both groups):  (a) 7; (b) 10  (data are from visual inspection of Figure 1 in the paper) | **‘Classic’ *method***  Not reported | No effects were tested |

**S1G Table Accuracy of peer review scoring reported in a 2-group study**

| **Study** | **Outcome** | **Innovation** | **Comparator** | **Effect** |
| --- | --- | --- | --- | --- |
| Sattler et al. 2015 | Percentage of reviewers who selected the correct NIH rating scale values | ***Reviewer training video***  74 | ***No-training group***  35 | Difference between groups P<0.05 (from linear regression) |

Familiarity with NIH rating scale was also reported for experienced and novice reviewers but only overall, not per study group, based on 5-point Likert-type scale. The difference between experienced/novice reviewers was tested statistically using mean and SD but this is inappropriate for ordinal data

**S1H Table Application preparation time reported in a single-group study**

| **Study** | **Outcome** | **Innovation aspects assessed** | **Effect** |
| --- | --- | --- | --- |
| Barnett et al. 2015 | Preparation time per proposal (for n proposals), days: (a) mean; (b) median (range) | ***Streamlined (short) proposal with accelerated peer review***  Funding round 1 (2012, n=74): (a) 6.8; (b) 5 (1 to 31)  Funding round 2 (2012, n=89): (a) 7.2; (b) 5 (1 to 30)  Funding round 3 (2013, n=34): (a) 7.2; (b) 5 (1 to 30)  Funding round 4 (2013, n=31): (a) 7.7; (b) 4 (1 to 48) | No effects were tested |

**EFFECTIVENESS OUTCOMES**

**S1I Table Funding decisions reported in 2-group studies**

| **Study** | **Outcome** | **Innovation, comparator and effect (where reported)** | | |
| --- | --- | --- | --- | --- |
| Gallo et al. 2013; Carpenter et al. 2015 | Percentage of proposals that shifted in either direction over the funding score threshold following peer review discussion | ***Teleconference***  (2 years: 2009-2010)  12.7 (of which 29.6% moved into the fundable range and 70.4% moved out of the fundable range) | ***Face-to-face meeting***  (2 years: 2011-2012)  10.0 (of which 34.6% moved into the fundable range and 65.4% moved out of the fundable range) | |
|  | Percentage% of proposals with (a) moderate/ high score changes and (b) low score changes over the funding score threshold in either direction | ***Teleconference***  (2 years: 2009-2010)  (a) 48.1  (b) 51.9 | ***Face-to-face meeting***  (2 years: 2011-2012)  (a) 69.2  (b) 30.8 | |
|  | Percentage% of proposals falling within the fundable range after peer review discussion | ***Teleconference***  (2 years: 2009-2010)  19.8 | ***Face-to-face meeting***  (2 years: 2011-2012)  15.4 | |
| Herbert et al. 2015  (authors were contacted and clarified interpretation of Table 3 in the publication) | Number (%) of proposals funded by each panel | ***7-member simplified panel***  Basic science (n=36): 8 (23)  Public health (n=36): 9 (25)  Total (n=72): 17 (24)  ***2-member ‘journal’ panel***  Basic science (n=36): 4 (12)  Public health (n=36): 2 (6)  Total (n=72): 6 (9) | ***12-person standard panel***  Basic science (n=36): 11 (31)  Public health (n=36): 4 (11)  Total (n=72): 15 (21) | |
|  | Funding agreement across panels, number (%) of proposals | ***Comparison of the two simplified panels***  *Both simplified panels funded:*  Basic science (n=36): 2 (6)  Public health (n=36): 2 (6)  Total (n=72): 4 (6)  *Only 7-member panel funded*:  Basic science (n=36): 6 (17)  Public health (n=36): 7 (19)  Total (n=72): 13 (18)  *Only 2-member panel funded*:  Basic science (n=36): 2 (6)  Public health (n=36): 0 (0)  Total (n=72): 2 (3)  *Not funded by either panel:*  Basic science (n=36): 26 (72)  Public health (n=36): 27 (75)  Total (n=72): 53 (74) | ***Comparison of standard vs 7-member simplified panel***  *Both standard and 7-member panel funded:*  Basic science (n=36): 4 (11)  Public health (n=36): 2 (6)  Total (n=72): 6 (8)  *Only standard panel funded*:  Basic science (n=36): 7 (19)  Public health (n=36): 2 (6)  Total (n=72): 9 (13)  *Only 7-member panel funded*:  Basic science (n=36): 4 (11)  Public health (n=36): 7 (19)  Total (n=72): 11 (15)  *Not funded by either standard or 7-member simplified panel:*  Basic science (n=36): 21 (58)  Public health (n=36): 25 (69)  Total (n=72): 46 (64) | ***Comparison of standard vs 2-member ‘journal’ panel***  *Both standard and 2-member panel funded:*  Basic science (n=36): 1 (3)  Public health (n=36): 0 (0)  Total (n=72): 1 (1)  *Only standard panel funded*:  Basic science (n=36): 10 (28)  Public health (n=36): 4 (11)  Total (n=72): 14 (9)  *Only 2-member panel funded*:  Basic science (n=36): 3 (8)  Public health (n=36): 2 (6)  Total (n=72): 5 (7)  *Not funded by either standard or 2-member simplified panel:*  Basic science (n=36): 22 (61)  Public health (n=36): 30 (83)  Total (n=72): 52 (72) |
|  | Agreement and disagreement in funding decision, % (95% CI) | ***Comparison of the two simplified panels***  *Agreement:*  Basic science (n=36): 78 (64-92)  Public health (n=36): 81 (67-92)  Total (n=72): 79 (68-89)  *Disagreement:*  Basic science (n=36): 22 (8-36)  Public health (n=36): 19 (8-33)  Total (n=72): 21 (11-31) | ***Comparison of standard vs 7-member simplified panel***  *Agreement:*  Basic science (n=36): 69 (56-83)  Public health (n=36): 75 (61-89)  Total (n=72): 72 (61-82)  *Disagreement:*  Basic science (n=36): 31 (17-44)  Public health (n=36): 25 (11-39)  Total (n=72): 28 (18-39) | ***Comparison of standard vs 2-member ‘journal’ panel***  *Agreement:*  Basic science (n=36): 64 (47-78)  Public health (n=36): 83 (69-94)  Total (n=72): 74 (62-83)  *Disagreement:*  Basic science (n=36): 36 (22-53)  Public health (n=36): 17 (6-31)  Total (n=72): 26 (17-38) |
| Mayo et al. 2006 | Agreement to fund, between ‘Ranking’ and ‘Classic’ PR approaches, number of proposals | ***‘Ranking’ vs ‘Classic’ method: new investigator proposals***  Agree to fund: 3  Disagreement: 4  Agree not to fund: 10  Total: 17  Kappa value: not reported | ***‘Ranking’ vs ‘Classic’ method: new team proposals***  Agree to fund: 3  Disagreement: 4  Agree not to fund: 8  Total: 15  Kappa value: not reported | ***‘Ranking’ vs ‘Classic’ method: all proposals***  Agree to fund: 6  Disagreement: 8  Agree not to fund: 18  Total: 32  Kappa value=0.36  (95% CI 0.02-0.70) |
|  | Estimated (theoretical) % of all possible reviewer pairings in ‘Ranking’ method in which the proposal would fail to meet the funding cut-off (shown for the 10 highest-ranked of each of 17 new investigator proposals and 15 new team proposals) | ***New investigator proposals:***  Rank 1 (top): 9  Rank 2: 29  Rank 3: 62  Rank 4: 56  Rank 5: 60  Rank 6: 74  Rank 7: 72  Rank 8: 84  Rank 9: 96  Rank 10: 87 | ***New team proposals:***  Rank 1 (top): 34  Rank 2: 27  Rank 3: 36  Rank 4: 40  Rank 5: 62  Rank 6: 84  Rank 7: 84  Rank 8: 94  Rank 9: 87  Rank 10: 96 | |

**S1J Table Funding decisions reported in single-group studies**

| **Study** | **Outcome** | **Innovation aspects assessed** |
| --- | --- | --- |
| Barnett et al. 2015 | Number (%) of eligible applications: (a) shortlisted; (b) interviewed; (c) funded | ***Streamlined (short) proposal with accelerated peer review***  Round 1 (2012; n=74): (a) 29 (39); (b) 11 (15); (c) 6 (8)  Round 2 (2012; n=89): (a) 26 (29); (b) 8 (9); (c) 5 (6)  Round 3 (2013; n=34): (a) 27 (79); (b) 11 (32); (c) 5 (15)  Round 4 (2013; n=31): (a) 22 (71); (b) 10 (32); (c) 5 (16) |
| Fleurence et al. | Numbers of proposals funded in phase 2 (scientist, patient and stakeholder PR) in relation to their ranking in phase 1 (scientist-only PR) | ***Phase 1 rank:*** ***Funded in phase 2 (total 25):***  1 to 25 13  26 to 50 8  51 to 152 4  ***Phase 1 rank:*** ***Discussed but not funded in phase 2 (total 73):***  1 to 25 11  26 to 50 12  51 to 152 50  ***Phase 1 rank:*** ***Not discussed in phase 2 (total 54):***  1 to 25 2  26 to 50 4  51 to 152 48 |

**S1K Table Process outcomes: views of peer reviewers (4 studies) or applicants (1 study)**

| **Study and outcome** | **Results** |
| --- | --- |
| Barnett et al. 2015  Views of applicants on the application process, provided by email | ***Applicants’ views on short proposals***  The authors reported several quotes of applicants’ opinions, all of which were positive and supportive of the short proposals; although one respondent is quoted as saying the 1,200-word limit was “challenging but not impossible”. It is unclear whether these are a complete reflection of all the applicants’ views. According to the quotes, applicants perceived the strengths of the innovation as being the simple online process, web-based seminar, reduction of unnecessary paperwork, and the focus on research ideas rather than applicants’ track records.  ***Applicants’ views on the feedback given to them***  The authors reported several quotes of applicants’ opinions, all of which were positive and supportive of the peer-review transcripts provided to applicants as feedback. It is unclear whether these are a complete reflection of all the applicants’ views. According to the quotes, applicants appreciated the quick feedback provided which was helpful for refining their proposals, and the learning opportunities for proposal development afforded by the feedback. The applicants’ views about transcripts appear to refer to only one of the four funding rounds studied (the first round in 2013), since transcripts were not provided to applicants in other rounds. The authors state that transcripts are time consuming (and therefore rarely provided to applicants in practice) and were not provided for the second funding round in 2013 due to time constraints. |
| Gallo et al. 2013; Carpenter et al. 2015  Views of reviewers (n=90), assessed in a survey (in 2012) after transition to teleconference panels, with answers scored on a scale of 1 (worst) to 5 (best). Also views of the funding agency. | ***Peer reviewers’ views:***  “To what extent did you find the panel discussions fair and thorough?’’ The average score for this question was 4.5 (98% of reviewers scored above 3.0) (98% of surveyed reviewers responded). The authors state that this question was also asked in a 2008 survey of this programme (when a face-to-face review setting was employed), and also gave an average score of 4.5.  ‘Thinking of your past experiences with in-person, on-site review panels, to what extent did the teleconference review panel format achieve a thorough review of each application?’’ The average score for this question was 4.0 (77% of reviewers scored above 3.0) (81% of surveyed reviewers responded).  The paper does not list all the questions that were asked, so it is unclear whether these are incomplete or selective results.  ***Funding agency’s views:***  The authors reported only that the feedback was positive and no change in review quality from the switch from face-to-face to teleconference reviews was noted (the funder is currently acquiring more survey data on this topic). |
| Fleurence et al. 2014  Views of reviewers (stated to be from focus groups and web-based surveys, but methods not reported) | ***Peer reviewers’ views:***  The authors summarise the themes that emerged from the survey and focus groups. Limited detail is given. The main findings were:  • Scientists’ appreciation of the perspectives offered by patients and stakeholders  • Recognition of a collegial and respectful process  • Challenges included: scientists had concern about non-scientists’ level of technical expertise and some non-scientists being considered less authoritative than scientists. Difficulties in understanding the unique PCORI review criteria (such as patient-centeredness)  • Suggestions for breaking down the hierarchy among reviewers included: alternating the order of oral presentation by reviewer types; adding a stakeholder or patient co-chair; and reducing the use of language that implies distinction (such as scientific and non-scientific).  The authors state that many patient reviewers requested more interaction with scientific reviewers before the in-person review panel (for example, in reviewer training or via e-mail). Furthermore, many reviewers suggested that PCORI use only 1 phase of review incorporating scientists, patients, and stakeholders.  The paper does not list all the questions that were asked, so it is unclear whether these are incomplete or selective results. |
| Holliday et al. 2010  Views of reviewers obtained after completion of the PR process (method of acquisition not reported) | ***Peer reviewers’ views:***  Question: Would more discussion between rounds be beneficial and, if so, would it have altered your final decision? Responses are reported from three or four of the five reviewers (unclear whether two of the responses are from the same or different reviewers). Two reviewers stated that a discussion would be helpful and two stated that a conference call would be helpful, but none stated whether it would alter their final decision.  Question: Did cumulative scoring result in the optimum outcome? Responses from three of the five reviewers are reported. One said ‘yes’. One felt that multiple rounds were not necessary since the leaders did not move very much between each round. The third reviewer said it depended on what was weighted more in each round, and commented that the round weighing scientific merit was omitted, so grants emphasised novelty over scientific merit.  Question: How did this process compare with more traditional scientific peer reviews and grant assessment processes? Responses from three of the five reviewers are reported. One said it was easier but another said it was more time-consuming, as they needed to reread proposals between rounds, suggesting one round may have been better. The third reviewer felt the process worked well, although they found the cumulative scoring novel.  Question: Would you recommend this process in the future to other funding schemes? Responses from three of the five reviewers are reported. They gave mixed but generally positive responses (one said ‘yes’, I think it is fair’; one said ‘maybe’ and felt the ranking approach to be helpful, and different to the process used in the study sections they had served on; and the third reviewer gave a narrative answer stating that the process emphasised novelty over scientific merit, which may be okay if that is what is intended).  The paper does not list all the questions that were asked, and responses from all 5 reviewers do not appear to be given for all questions, so it is unclear whether these are incomplete or selective results. |
| Vo et al. 2015  Reviews of reviewers (n=110) obtained by 10-item questionnaire after the peer review meeting session | ***Peer reviewers’ views:***  Answers for six of the 10 questions asked in the questionnaire are tabulated in the paper. Overall, peer reviewers rated virtual peer review positively in terms of the questions ‘Easy access’, ‘Pre-meeting session useful’, ‘Good display’ and ‘Good audio’ (67% to 90% said ‘yes’ to these four questions, 5% to 26% said ‘no’, and 0% to 7% did not respond, although overall there was a low response rate). However, answers to the remaining two questions ‘Technical difficulties’ and ‘Would use virtual review again’ indicated that 26 % experienced technical difficulties and 33% would not use virtual peer review again. The paper states that Special Emphasis Panel members were ‘more receptive than Study Section members to this new technology (75 % vs 42 %, respectively; P<0.05)’, but does not state which question(s) these data refer to or which statistical test was employed.  There are some uncertainties: it is not reported which study sections or panels the reviewers were from; and the question about ease of access appears to have required responses on a 5-point Likert-type scale, but it is unclear how the proportions of yes/no answers were determined from this.  The paper states that virtual reviewers noted several advantages to the web-based sessions, including less travel (64%), decreased costs (19%) and faster reviews (10%). The disadvantages mentioned were minimal interaction among reviewers (43%), distractions (14%) and less thorough reviews (14%).  In the discussion, the paper refers to a specific difficulty encountered by the peer reviewers which was that they believed they were unable to focus on the small computer screen for a long period of time. The authors suggest that this problem could be minimised if breaks are scheduled frequently (they suggest for, example, a 10-minute break for every 90-minute session). However, the authors suggest that to avoid this problem, any planned 2-day peer review session should be held as a face-to-face meeting. Improvements to the technology could include making the video display occupy a full computer screen.  It is unclear why the paper does not report responses for four of the questions asked. |
